# Perrotta Integrative Clinical Interviews‐3 (PICI‐3): Development, regulation, updation, and validation of the psychometric instrument for the identification of functional and dysfunctional personality traits and diagnosis of psychopathological disorders, for children (8–10 years), preadolescents (11–13 years), adolescents (14–18 years), adults (19–69 years), and elders (70–90 years)

**DOI:** 10.1002/ibra.12148

**Published:** 2024-02-13

**Authors:** Giulio Perrotta

**Affiliations:** ^1^ Istituto per lo Studio delle Psicoterapie (ISP) Rome Italy

**Keywords:** dysfunctionality, functionality, Perrotta Integrative Clinical Interviews‐3 (PICI‐3), personality traits, psychopathological diagnosis

## Abstract

The Perrotta Integrative Clinical Interview, second version (PICI‐2) requires structural and functional updates, based on clinical and academic experience, especially in terms of functional traits and interpretation of psychopathological disorders. The Perrotta Integrative Clinical Interviews‐3 (PICI‐3) was created and structured into four sections, dedicated to dysfunctional traits in children and pre‐adolescents (PICI‐C‐3, 8–13 years) and in adolescents, adults, and the elderly (PICI‐TA‐3, 14–90 years), to common secondary disorders (PICI‐DS‐3, 8–90 years) and functional traits (PICI‐FT‐3, 8–90 years), with the identification of all functional elements and structural aspects of personality according to the model underlying the PICI (IPM). Selecting 1732 subjects, between 8 and 90 years old, the statistical analysis showed that the psychometric test has a well‐defined and stable construct, with the variables well represented and positively correlated with other constructs already validated. In particular: (a) the PICI‐TA‐3 (Section A) was compared with the Minnesota Multiphasic Personality Inventory‐2‐Restructured Form (MMPI‐2‐RF), obtaining 99.3% compatibility of results, with a Pearson's coefficient (*R*) of 0.999 and *p* < 0.001; (b) the PICI‐C‐3 (Section B) was compared with the Child Behavior Checklist (CBCL), obtaining 94.1% compatibility of results, with a Pearson coefficient (*R*) of 0.969 and *p* < 0.001; (c) the PICI‐FT‐3 (Section D) was compared with the Big Five Personality Test (Big5), obtaining 89.4% compatibility of results, with a Pearson coefficient (*R*) of 0.797 and *p* < 0.001. The PICI‐3 is a valid, efficient, and effective psychometric tool to identify the functioning or dysfunction of personality traits for psychopathological diagnosis.

## Graphical abstract

The psychometric instrument Perrotta Integrative Clinical Interviews‐3 (PICI‐3), which is efficient, effective, and valid, aims to identify functional and dysfunctional personality traits, in children (8–10 years), preadolescents (11–13 years), adolescents (14–18 years), adults (19–69 years), and the elderly (70–90 years), with the aim of ensuring the therapist's precise psychological and psychopathological framing of the patient, at the exact time of questionnaire administration, in relation to both structural and functional elements, so that the clinical strategy can be organized while minimizing interpretation errors

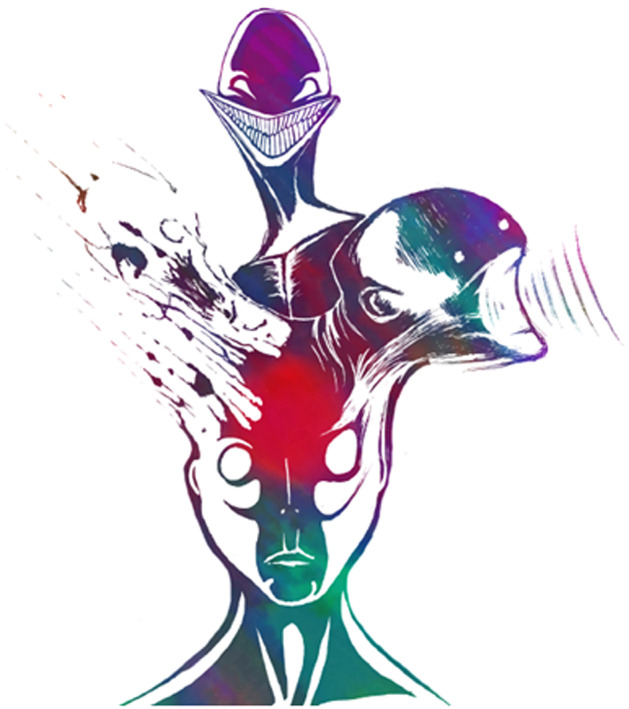

## 1 BACKGROUND

### 1.1 The concept of “personality”: Introduction and historical background

The definition of “personality” has always been debated and not universally agreed upon in the literature, as it pertains to the different models proposed and feeds on its different nuances. Hippocrates tried to explain it using the concept of humor (melancholic, choleric, phlegmatic, and sanguine types); S. Freud anchored it to the construct of internal instances (Ego, Ex, and Superego) according to five different models (topographic, dynamic, economic, genetic, and structural); C. G. Jung considered it the synthesis product of the individual and collective history of the person, recognizing eight different types (reflective‐extroverted, reflective‐introverted, sentimental‐extroverted, sentimental‐introverted, perceptual‐extroverted, perceptual‐introverted, intuitive‐extroverted, and intuitive‐introverted); H. Eysenck, on the other hand, was the first to define the personality of the individual according to a general concept, labeling it as “the stable and enduring organization of a person's character, temperament, intellect, and physique, an organization that determines his or her full adaptation to the environment” and determining the four levels of personality organization arranged hierarchically—(1) the three personological dimensions, such as extroversion‐introversion, neuroticism and psychoticism; (2) the trait configuration, understood as stable characteristics of conduct; (3) the recurrent responses, understood as actions that are frequently repeated and create patterns of behavior; (4). the specific and occasional responses, which do not necessarily have the character of stability or are indicators of personality)—a model later expanded by R. B. Cattel who identified 16 personality factors, by G. Allport who theorized the concept of personality traits in a more structured way, distinguishing them into cardinal (most characterizing because they are strong and pervasive), central (in that they capture the essence of the individual) and secondary (in that they define the specificity of the personality in its totality), and by McCrae‐Costa who identified five specific global personality traits on which their entire model was built (extroversion, agreeableness, conscientiousness, neuroticism, and openness to experience). Other important contributions also came from H. Murray and A. Maslow, on the subject of the theory of needs and the hierarchical pyramid of needs, and from O. Kernberg, on the subject of psychopathological diagnosis (for him the psychopathology of personality is determined by the psychic structures resulting from affective experiences with primary significant objects, distinguishing the three forms of organization such as neurotic, borderline, and psychotic).^1^, ^2^, ^3^, ^4^, ^5^, ^6^, ^7^, ^8^, ^9^, ^10^, ^11^, ^12^

### 1.2 Integrative Psychodynamic Model (IPM)

Based on these assumptions, the first model (Integrative Psychodynamic Model, IPM) was created, taking into account the following structural elements^1^, ^13^, ^14^, ^15^, ^16^:

1) The “*personality*” is an organized and structured, stable and enduring complex of specific characteristics, and a distinction must be made between personality and personality trait, and again between temperament and character, as well as between constitution, behavior, habit, attitude, aptitude, inclination, and predisposition (Table 1).
2) The “*personality*” is made up of a structural part (the structural scaffolding) and a functional part (how the person functions, concerning himself or herself, and the environment of reference). The “personality structure” is thus the set of a person's deep and stable, largely unconscious, personal psychological characteristics that are expressed in every aspect of one's daily life. Such a structure makes each unique and unrepeatable and consists of two parts: (a) one plastic and modifiable (composed of the external reinforcers that design attitudes, aptitudes, inclinations and predispositions), in continuous interaction with the external environment that influences it, to make it adherent and appropriate to contingent situations; (b) another fixed, rigid, enduring and stable (composed of temperament, character, and intelligence), which comprises the biological structure and learnings firmly acquired and permanent over time. “Personality functioning,” on the other hand, pertains to personological dynamics, both as a self‐referential system (capable of self‐reflection to achieve goals and comply with norms) and as a self‐regulatory system (enactment of strategies that ensure the ability to adapt to the environment and modify it by the satisfaction of one's needs). The way a person interacts with the environment and constructs his or her identity also depends on an individual's motivations and causes concerning his or her needs and wants. Functioning is assessed on an increasing scale of dysfunctional (or maladaptive concerning the environment) traits present in one's personality framework (healthy, neurotic, borderline, and psychotic).
3) Underlying PICI‐1 is a specific model named “*Integrated Psychodiagnostic Model*” (*IPM*), which meets all the needs of updating the previous psychodynamic, cognitive‐behavioral, and constructivist strategic models through some adjustments made (Tables 2 and 3):

a) *First corrective*: Taking the Freudian model of the first topical (Conscious, Unconscious, and Preconscious) as the basis, the structural modification is made on the preconscious, making it a function of the conscious; in fact, the “topographical tripartite model” becomes “binary‐structural” (conscious‐unconscious).
b) *Second corrective*: Taking the Freudian model of the second topical (Ego, Ex, and Superego) as the basis, the structural modification is made on the Superego, making it become a function of the Ego; in fact, the “tripartite topographical model” becomes “binary‐functional” (Ego‐Ex).
c) *Third corrective*: Taking Jung's model of psychic structure as the basis, the collective instances of the unconscious are imported; in fact, the “Jungian psychic model” becomes an enhancement of the “binary‐structural” component of the new model (IPM). In this sense, the Ego performs the functions of: (a) mediation and filtering by defence mechanisms, and by guilt and shame, on the instincts of the Ex; (b) preservation, maintenance, and reenactment of unremembered memories, called “Person”; (c) relational contacts with the external environment, using perceptions, emotions, and feelings, through the use of the mask, called “Character”; (d) contacts with the Ex, through the boundary line that divides them (and never directly) called “Self” (exactly the opposite of Jungian theorizing, which considers the ego a part of the Self). Again, the Ex performs the functions of: (a) preservation and maintenance of removed personal memories, partly inaccessible; (b) preservation and maintenance of destructive drives and energies, totally inaccessible, called the “Shadow”; (c) preservation and maintenance of ancient energies, stemming from an ancestral past (identified with the collective unconscious and the biological matrix of the family tree), called the “Past.”
d) *Fourth corrective*: The functional component of the new model (IPM) is enriched with the theorizing of O. Kernberg, which, in addition to assessing attachment and affectivity profiles, is corrected by providing for an analysis of the following specific parameters in functional personality investigation: (a) ancestral investigation (family relationships and family history); (b) investigation of childhood attachment and object relations; (c) study of individual identity, the robustness (or strength) of the ego, and its subjective emotional language; (d) evaluation of defensive organization; (e) evaluation of reality examination and study of the Self and Superego.

**Table 1:** Structural definitions of Perrotta Integrative Clinical Interview‐1 (PICI‐1).

Structural element	Definition
*Personality*	Organized and complex, stable and enduring set of psychic characteristics, behavioral and relational patterns that define the person, taking into account the environment of reference
*Personality trait*	A constant personality trait, understood as a specific cognitive‐behavioral mode, capable of modifying the subject's natural perceptual‐emotional state, toward himself and the environment with which he is relating
*Temperament*	Innate personality component, about genetic predisposition and stimuli learned during gestation (structural‐relational dimension)
*Character*	Modulated personality component, acquired through stimulus learning in early life and affected by temperament (which is called “disposition” here) and conformity or nonconformity to social values and standards (structural‐emotional dimension)
*Intelligence*	Complex psychic and mental faculties that, through cognitive processes (such as learning, memory, reasoning, comprehension and reflection, including the skills of logic, abstraction, planning, creativity, critical thinking, and problem solving), enable one to understand concepts and organize one's behavior accordingly, both concerning the ideational and realization stages, to achieve a specific goal in the shortest possible time (structural‐cognitive dimension)
*Constitution*	Outward, the physical and anatomical configuration of the person, the result of innate tendencies and learned behaviors until adolescence
*Behavior*	Set of expressed behaviors that are nurtured by the constellation of personality traits, whether functional or dysfunctional, in adaptive or maladaptive response to the reference environment
*Behavioral habit*	Specific reinforcement of a specific type of behavior, repeated over time because it is structured in a complex of actions aimed at satisfying a specific need or necessity
*Attitude*	Cognitive‐behavioral mode that is positively conditioned by the environment to which it refers and that predisposes it to certain repeated actions over time, by a specific personality trait
*Aptitude*	Reinforced cognitive‐behavioral mode, a consequence of the effects of the summation of two personality traits of the same characterization
*Inclination*	Enhanced cognitive‐behavioral mode, a consequence of the effects of the summation of three personality traits of the same characterization
*Predisposition*	Established cognitive‐behavioral mode resulting from the effects of the summation of four personality traits of the same characterization

**Table 2:** Structural and functional correctives brought by the model to major psychodynamic theorizing on psychic instances.

Structural element	Definition
*Basic structural model*	S. Freud's first topical (preconscious, conscious, and unconscious) → the preconscious becomes a function of the conscious (*binary‐structural assumption*)
*Adjustment to the basic structural model*	Psychic model of C.G. Jung →
	a) The Ego mediates and filters by defence mechanisms the drive instincts of the Ex, preserves, maintains, and evokes unremembered memories, using the “Person” function, receives external stimuli using sensory state and perceptions, using the “Character” function, and maintains contacts with the Ex, using the boundary line that divides them (and never directly) using the “Self” function. b) The Ex retains and maintains removed personal memories and retains and maintains destructive drives and energies, all using the “Shadow” function, while it retains and maintains ancient energies and memories, stemming from an ancestral past (identified with the collective unconscious and the biological matrix of the family tree) using the “Past” function.
	(*enhanced assumption of the binary‐structural*)
*Basic functional model*	S. Freud's second topic (Ego, Ex, and Superego) →
	The Superego becomes a function of the Ego
	(*binary‐functional assumption*)
*Adjustment to the basic functional model*	O. Kernberg theorizes →
	Functional investigation of the personality profile must take into account ancestral investigation (family relationships and family history) (1), characteristics of childhood attachment and object relations (2), the study of individual identity and thus the robustness of the ego and its subjective emotional language (3), defensive organization (4), and reality examination and the study of the Self and Superego (5)
	(*enhanced assumption of the binary‐functional*)

**Table 3:** Scheme of the functions of psychic instances according to the new model.

Psychic instance	Definition
*EGO*	It is the antagonistic instance of the Ex, totally conscious. It is an endowment present at birth but during the first 2 years of the individual's life, it strengthens until it finds its dimension (slightly larger than the Ex, in the absence of psychopathological conditions). It is manifested externally through the “Person,” which in turn masquerades through the “Character” function. The Ego has two main functions in interacting with the unconscious world: (a) the “Self” and the “Superego” (through the “defence mechanisms”). The Self, which is formed after the first year of life, creates a clear separation from the unconscious world to contain it. While it must contain it, on the other hand, it allows the transition to the Ego through the defence mechanisms of the Superego, which act as real energy filters.
*ID or EX*	It is the main instance par excellence; it is the endowment operating system from birth. During the first year of life, it surrenders part of itself to the conscious plane for it to develop. It is in constant contact with the pro‐deepest parts and acts as the containing anti‐chamber of the “Shadows” (the actual container of drive and destructive energies, governed by the domination of the egoistic and individual pleasure principle) that feed from the “Past” (of collective memories and ancestral memories of forbidden access to the conscious).

**Figure 1:**
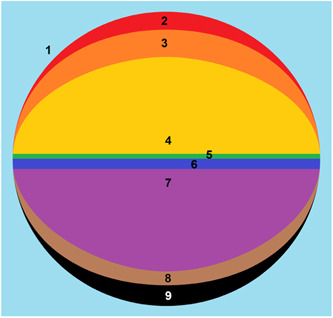
Graphic representation of the psychic instances, according to the new model (IPM). In detail: 1. Outdoor Environment 2. Character (external physical body) 3. Person (constitution‐internal physical body, intelligence‐mental body, character‐emotional body and temperament‐spiritual body) 4. Ego (etheric body) 5. Superego (social body) 6. Self (causal body) 7. Id or Ex (soul body) 8. Shadow (dark body) 9. Past (ancient or collective body). [Color figure can be viewed at wileyonlinelibrary.com]

Graphically, the new model (IPM) is represented as follows (Figure 1) in all its instances:

1) The light blue color represents the *External Environment*.
2) The red color represents the character, understood as the social representation of the Person (or “*physical body*”).
3) The orange color represents the *Person*, understood as the synthesis of the Ego and the mediation process with the Ex through the Superego and Self functions. According to this view, the four dimensions of Person are: intelligence represents the cognitive dimension (or “*mental body*”), which makes use of cognitive functions to process external reality and adapt as best as possible; character represents the emotional‐affective dimension (or “*emotional body*”), which feeds on desires, needs, and wants; temperament represents the intimate and relational dimension (or “*spiritual body*”), which feeds on emotions and feelings; constitution represents the physical dimension (or “*physical body*”), which is represented by the Person (or Person masks).
4) The color yellow represents the *Ego* (or “*etheric body*”).
5) The green color represents the *Super‐Ego function* (or “*social body*”), which uses defence mechanisms to filter out instances coming from the Ex and already partially depotentiated by the Self.
6) The blue color represents the *Self* (or “*causal body*”), understood as the function of the Ego and the wall of separation between conscious and unconscious, filtering for instances coming from the Ex. It limits and depotentiates the pleasure principle, effectively containing the Shadow and the Past.
  Above the blue line is the conscious plane (*Conscious*); immediately below begins the unconscious plane (*Unconscious*).
7) The color purple represents the *Ex* (or “*soul body*”), the container of memories that have been removed but can still be accessed by certain hypnotic induction techniques.
8) The color brown represents the *Shadow* (or “*dark body*”), the container of the most destructive energies and drives.
9) The color black represents the *Past* (or “*ancient body*”), the container of the collective Unconscious that communicates with the Shadows through the archetypes.

### 1.3 Psychodiagnostic Investigation Model (PIM‐1)

Based on the IPM, a second model (*Psychodiagnostic Investigation Model, PIM*), entirely devoted to the restructuring of psychopathological disorders and their clinical diagnosis, was combined. The “psychopathological disorders” (or psychopathologies), in this new model, are the product of the structural and functional alterations of the instances contained in the model itself, in response to the external (educational and social) environment, but in terms of different from the classical and/or modern psychodynamic model (hypertrophic Ego‐hypotrophic Ex/hypotrophic Ego‐hypertrophic Ex); in this module, attention will instead be paid exclusively to “ego‐functions,” as physically the Ego and the Ex remain structurally unchanged. Therefore, three relevant psychodiagnostic distinct hypotheses may be tested:

A) Ego functions (Superego/Self) are hypereactive (Superego +/Self +). Their functions of filtering (Self) and energy depletion (Superego) turn out to be more intense and powerful than necessary, and the functional mechanism of the Ego is “hypervigilant.” The Ex consequently experiences energy depletion. In this hypothesis, we see the onset of the psychopathological conditions classified as neurotic (cluster A, according to the new classification).
B) Ego functions (Superego/Self) are unstable (Superego +/Self ‐ or Superego ‐/Self +). Their functions of filtering (Self) and depowering energy (Superego) turn out to be oriented toward an overall functional weakness of the Ego, which is therefore “fragile.” The Ex consequently has a greater chance of allowing more enhanced energy to filter through to the conscious level. In this hypothesis, we see the emergence of the psychopathological conditions classified as borderline (or at the limit, cluster B, according to the new classification).
C) Ego functions (SuperEgo/Self) are shattered (SuperEgo/SELF). Their functions of filtering (Self) and depowering energy (SuperEgo) turn out to be oriented toward a full functional weakness of the Ego, which is therefore “fragmented.” The Ex consequently has a total ability to let the enhanced energy filter down to the conscious level. In this hypothesis, we see the onset of the psychopathological conditions classified as psychotic (cluster C, according to the new classification).

Based on the new model (PIM), however, it was realized that even psychopathological investigations had to completely change focus, for if everything is “personality” and not just a simple stable and enduring representation, it seems clear that a diagnosis of a psychopathological condition such as anxiety or obsessive disorder necessarily had to be framed within a new theorization and classification of personality disorders (whereas until now personality disorders have always been distinct from other psychopathological disorders, possibly connected by clinically relevant comorbidities), taking into account not only categorical and structural profiles but also and above all functional, dynamic, and neurobiological.

The new model, therefore, must take into account the following assumptions:

a) The psychopathological diagnosis is always “personological” and always refers to a habitual, stable, persistent, and pervasive pattern of experiences and behaviors that deviates significantly from the culture to which the individual belongs and is manifested in at least two areas among cognitive experience: (i) affective experience (ii) interpersonal functioning and impulse control. “Personological diagnosis” can be made from the age of 12 years, while for patients below the threshold, the diagnosis is always “psychopathological presumption of personality,” still deserving of clinical treatment if the number of dysfunctional traits and/or behaviors found cause significant abnormalities such as to merit intervention. They will be referred to, in these cases, not as personality disorders but simply as “specific disorders” (in that they lack the requirement of stability, in a personality that is not yet perfectly structured) and will follow a precise nosographic categorization of them that tends to differ from true personality disorders. In adolescents and adults, however, each diagnosis is framed within a precise personological framework that defines the specific personality disorder, according to the nosographic list.
b) Each personality disorder is described in its nine core characteristics, referred to as “dysfunctional personality traits,” and to be diagnosed it must exhibit five or more specific traits of the same personality disorder, in a dysfunctional personality pattern that is habitual, stable, persistent, and pervasive, according to a scale ranging from mild (or oriented, with five traits), significant (or sensitive, with six traits), moderate (or vulnerable, with seven traits), severe (or impaired, with eight traits), and extreme (or severely impaired, with nine traits). To be considered a “dysfunctional trait,” however, the symptomatology must have persisted for at least 3 continuous months, otherwise one must speak of “dysfunctional behavior,” and this circumstance will not contribute to the diagnosis of a personality disorder, although it may still be worthy of psychological support.
c) The psychopathological diagnosis must take into account the definition of the primary disorder (the one with more dysfunctional traits of the same type) and the secondary disorders that feed into the primary dysfunctions (dysfunctional behaviors, attitudes, inclinations, and predispositions), in addition to the 12 categories of disorders attached to the personological framework (taken from the Diagnostic and Statistical Manual of Mental Disorders, DSM‐V‐TR^17^), such as (a) neurodevelopmental disorders; (b) brief or acute psychotic disorder; (c) catatonic disorder; (d) selective mutism; (e) nutritional disorders; (f) evacuation disorders; (g) sleep‐wake disorders; (h) gender identity disorders; (i) paraphiliac disorders; (j) sexual dysfunction disorders, in the absence of organic basis; (k) drug and/or comfortable addiction disorders; (l) suicidal tendency. The disorder with the most dysfunctional traits represents the principal diagnosis, while all other disorders that have at least five traits represent the representative trait connotation (e.g., in a patient with seven anxiety traits, five phobic traits, and four obsessive traits, the principal diagnosis will be “personality anxiety disorder, with phobic traits,” while the four obsessive traits will not be reported but will serve the therapist to build a more needs‐centered psychotherapeutic work for the patient, working on the obsessive components as well). The traits of other disorders that best define the main disorder must numerically be the most other of all the disorders present in the chart; if there are possibly at least four dysfunctional traits present in other disorders, these should be considered as “psychopathological features” worthy of in‐depth clinical. The absence of psychopathological traits or otherwise a value of less than 2/9 by type is equivalent to a diagnosis of a “healthy subject.”
d) Taking cues from the Diagnostic and Statistical Manual of Mental Disorders (DSM‐V‐TR)^17^ and the Psychodynamic Diagnostic Manual (PDM‐2),^18^ the models underlying the PICI (IPM and PIM) identified 25 different possible structural categories of dysfunctional personality, with their respective characteristics, for the adolescent and adult population aged 12 years and older (PICI‐1TA) (Supporting Information 1), and 19 different possible structural categories of dysfunctional personality, with their respective characteristics, for the child population aged 4–11 years inclusive (PICI‐1C) (Supporting information 2).

### 1.4 Perrotta Integrative Clinical Interview, first version (PICI‐1)

Based on the previously described models, the first two interviews (PICI‐1C for the population aged 4–11 years and PICI‐1TA for the population aged 12 years and older) were constructed according to the clinical model of the Diagnostic and Statistical Manual of Mental Disorders (DSM‐V‐TR)^17^ and the Psychodynamic Diagnostic Manual (PDM‐2).^18^ The first version of the “1TA” (adolescent and adult) interview was structured in 195 items (173 dedicated to personality disorders and 22 dedicated to common secondary disorders) with YES/NO response, referring to the last trimester of life, while the first version of the “1C” (children) interview was structured in 150 items (128 dedicated to personality disorders and 22 dedicated to common secondary disorders) with YES/NO response, referring to the last trimester of life; the affirmative response to the specific item activates the corresponding dysfunctional trait(s). For diagnosis, an absorption grid was then applied among the specific psychopathological categories. Some technical refinements were then incorporated throughout the publication.^19^

Subsequent research compared the PICI‐1 with the Minnesota Multiphasic Personality Inventory (MMPI‐II),^20^ in a study of a sample of 472 subjects, demonstrating a diagnostic reliability of 98.73% concerning the diagnoses obtained from the Minnesota test's integrated clinical interview, and with a greater indication of the dysfunctional traits to be treated in psychotherapy. However, both during the drafting of the PICI‐1 and during the elaboration of the aforementioned research, some critical issues emerged referring precisely to the neurotic profiles: in fact, it was noted that, in the presence of symptomatological hyperactivation, a whole series of traits referable to different disorders were active, in addition to the five dysfunctional traits for each category. This inconsistency was analyzed and addressed during the final diagnosis, but it suggested making some structural and functional corrections to the PICI‐1.^21^

### 1.5 The structural and functional changes to PICI‐1 (PICI‐1‐v2)

Based on specific clinical observations evident in the previously narrated research, the following corrections to the basic PICI‐1 model have been suggested. At the diagnostic level: (1) the diagnosis should take into account the first two highest levels of dysfunctional traits, considering the next three lower levels as items of psychotherapeutic interest. In the hypothesis of dysfunctional overactivation, the diagnosis should be reevaluated at the end of the psychotherapeutic course, because the outcome of the test could be distorted; (2) the diagnosis should take into account, in its final formulation, the primary disorder (P, principal diagnosis), co‐primary disorders (M, mixed diagnosis), comorbidities (C), secondary disorders, (S) and tertiary traits (T); (3) on symptomatic persistence of symptoms and personality plasticity, mindfulness strategies and techniques can facilitate evolutionary change in therapy, provided that the patient's motivation is real, concrete, and current and that the complained of dysfunctional traits are not excessively disabling, there is a need for drug therapy and/or have been present for more than 1 year); (4) about absorptions between psychopathological classes, anxiety disorder absorbs somatic disorder, phobic disorder, and manic disorder, the latter becoming specific traits of (main) anxiety disorder, while psychotic disorders absorb all other neurotic disorders.^22^, ^23^

### 1.6 Perrotta Integrative Clinical Interview, second version (PICI‐2)

The dedicated test for children (PICI‐2C) remained unchanged, while the second version of the test (PICI‐2TA)^24^ was modeled by making the following changes:

1) The concept of ego synchrony of personality disorders is mitigated by the model underlying the PICI, and therefore the possible presence of ego synchrony in the patient concerning his or her symptoms is discernible only when the psychopathological impairment is severe (>7/9 dysfunctional traits), while in all other cases, it is possible to witness egodystonic patients, concerning their symptoms, while still having values relevant to the diagnosis of personality disorder.
2) Although six different personality disorders are distinguished in the test, the psychopathological classes afferent to the neurotic area must be treated as if they were a unitary disorder^25^ (referred to as “*neurotic personality disorder, NPD*”) (Supporting information 3). For the child population, the same structure can be applied, however, considering it simply as “neurotic disorder.”
3) The complexity of the psychopathological diagnosis of borderline disorder and bipolar disorder must be addressed during therapy and mediated by the responses obtained during psychotherapy, since manic and depressive traits induce the activation of bipolar and borderline traits, as they are common. Indeed: in the bipolar patient, there is a marked tendency for emotional and mood instability, which is more rigid than that of the borderline and is enriched by irrational beliefs taken to extremes, between depressive and manic episodes representing the patient's two main modes (albeit with four different possible sub‐hypotheses), however, depressive episodes are common to depressive disorder, while manic episodes are common to borderline disorder and some psychotic activations; in the borderline patient, there is a greater and more pronounced tendency toward impulsivity and aggression, with more emotional and affective fluctuations (as it is a relationship disorder), sudden and rapid, and depressive and manic episodes that are shorter and more circumscribed in the relevant timeframe than in the bipolar. It is, therefore, more than correct to separate the two interpretative hypotheses, precisely because of their peculiar characteristics of the behavioral and emotional‐affective picture, although they have several commonalities that would seem to overlap. After the item assignments with the specific dysfunctional traits, the following corrections should also be made at the diagnostic confirmation stage (Supporting information 4).
4) The sadistic‐masochistic and narcissistic (overt‐covert) scales seem to influence each other, and therefore during the clinical interview, it is also useful to subject the patient to psychometric tests assessing the individual sexual matrix^26^ and sex‐related profiles.^27^, ^28^, ^29^, ^30^, ^31^, ^32^, ^33^, ^34^, ^35^, ^36^, ^37^, ^38^, ^39^, ^40^, ^41^, ^42^, ^43^, ^44^, ^45^, ^46^, ^47^, ^48^, ^49^, ^50^, ^51^, ^52^, ^53^, ^54^, ^55^, ^56^, ^57^, ^58^, ^59^, ^60^, ^61^, ^62^, ^63^, ^64^, ^65^, ^66^, ^67^, ^68^, ^69^, ^70^, ^71^, ^72^, ^73^, ^74^, ^75^, ^76^, ^77^, ^78^, ^79^, ^80^, ^81^, ^82^, ^83^, ^84^, ^85^, ^86^, ^87^, ^88^, ^89^, ^90^, ^91^, ^92^
5) The items were reduced to 173, with the remaining 22 items being separated, as the common secondary disorders deserve more in‐depth investigation during the clinical interview.
6) The questionnaire dedicated to functional personality traits (PICI‐2FT), structured in 18 items, on a L0–4 scale, was introduced.
7) the administration of the PICI‐2 takes a picture of the patient's historical moment and not the previous one; therefore, it may be the case that some results are biased or distorted by the positive or negative historical moment the patient is experiencing, and it is up to the therapist to clearly and comprehensively frame the patient's anamnestic universe to understand any overactivation or omission of activation following a moment of stability of the patient, which in reality hides the true extent of his or her clinical manifestation. With these correctives, the PICI‐2TA was administered to 718 participants and compared again with the MMPI‐II,^24^ proving valid, efficient, and effective in 99.7% of the comparisons, while the remaining 0.3% seemed to be attributable to circumstances identifiable as the interpretive limitations; for reasons of structural diversity, it was not possible to compare the test related to functional traits (PICI‐2FT).

### 1.7 Aim

A validation study was conducted to determine whether the proposed psychometric instrument (PICI‐3) is capable of being reliable, efficient, effective, and valid for the identification of the subject's functional and dysfunctional personality traits to assess his or her personality profile. The purpose of the present discussion is to try to determine whether, in the current state of scientific knowledge, it is possible to validate the proposed psychometric instrument about the specific topic, according to the basic model used.

## 2 MATERIALS AND METHODS

### 2.1 Study design

Development, adjustment, updates, and validation of a psychometric instrument able to identify and assess personality structure and function, based on the reference model, third version (PICI‐3).

### 2.2 Materials and methods

Concerning the materials used, the PICI in its third version (PICI‐3) was compared during validation with the MMPI‐II (for the part related to the adolescent, adult, and elderly population), Child Behavior Checklist‐CBCL (for the part related to the child population) and Eysenck Personality Questionnaire‐EPQ (for the functional trait test, FT). The third version of the test (PICI‐3) was remodeled with the changes reported here:

1) STRUCTURAL INTERVENTIONS (static elements):

a) The PICI, in its third version, is structured into four different sections (A, B, C, D) that are complementary, to profile the personality framework as a whole. Section A (Supporting Information 5) is devoted to items (173 with responses on a yes/no scale) that detect the possible presence of dysfunctional personality traits (both in their structural and functional components), for adolescents (14–18 years), adults (19–69 years), and the elderly (70–90 years); section B (Supporting Information 6) is devoted to items (128 with yes/no scale responses) detecting the possible presence of dysfunctional traits (both in their structural and functional components), for children (8–10 years) and preadolescents (11–13 years); section C (Supporting information 7) is devoted to items (12 with multiple type‐specific responses) that detect the possible presence of psychophysical secondary disorders, for all developmental ages (8–90 years); section D (Supporting Information 8) is devoted to items (24 with responses on an L0‐5 scale) that detect functional personality components, for all developmental ages (8–90 years).
b) Numerous items, in their grammatical and syntactic formulation, were modified, without changing the original meaning, but contextualizing it with appropriate clarifications that were still made during the clinical interview and interview administration.
c) For numerous items, for the dysfunctional trait interviews, the item assignments dedicated to specific individual traits were modified for both Section A (Supporting information 9) and Section B (Supporting information 10).
d) Compared with the previous version (PICI‐2), the new version (PICI‐3) includes a third interview (section C) entirely devoted to the investigation of psychophysical secondary disorders, previously left to the free investigation of the therapist in the clinical interview; in this version, the individual categories and subcategories are specified, with a section also devoted to other morbid conditions that are nevertheless relevant, consistent with the symptoms and categories described in DSM‐5.
e) Compared with the previous version (PICI‐2), the new version (PICI‐3) restructures the interview devoted to functional traits (section D), previously left to a simple parameter assessment for the 18 areas of investigation. The areas are expanded from 18 to 24, and each includes five different behavior styles (each with a score to be assigned with an L0‐5 scale), to assess the specific functioning of all investigated components.

2) FUNCTIONAL INTERVENTIONS (interpretive elements):

a) Section A (PICI‐TA‐3) remains dedicated to dysfunctional personality traits, in adolescents, adults, and the elderly, restringing the child registry age from 12 to 14 and extending the adult registry age up to 90, by the population sample selected for this validation study. The structure remains the same, with yes/no responses, but the time reference is extended to the last quarter (unlike previous application versions that referred to the last bimonth), administered by specialized staff, in support of the patient. On the subject of data interpretation, the PICI‐TA‐3 modifies the assignments of some items and their specific dysfunctional trait activation values, avoiding more errors of symptomatological or diagnostic overlap, during the clinical interview; for the same reason, the assignment codes of the absorbing (able to absorb) and absorbed (able to be absorbed) categories have also been revised. Finally, it is officially established that the diagnosis in the PICI‐TA‐3 is both structural (relating to the category most representative of dysfunctional traits) and functional (relating to the representative categories immediately following the first); in this sense, the first category represents the dysfunctional structure of reference, while the subsequent categories define the pathological functioning of the subject, about the structure identified. The covert category is definitively separated from the overt, in narcissism, thus becoming 25 categories and no longer 24 (previously, category No. 13 was separated into “13a” and “13b”).
b) Section B (PICI‐C‐3) remains devoted to dysfunctional traits, in children and preadolescents, restringing the child registry age from 4 to 8 and extending the opposite registry age up to 13 (whereas in the past the limit was 4–11 years) years, by the population sample selected for this validation study. Thus, it is argued that it is not possible to speak of personality structure and functioning in subjects younger than age 14, while it is consistent to speak of “psychic internal functioning” since the symptomatology suffered may not necessarily be capable of impacting the personality and thus remain anchored throughout the subject's life; moreover, unlike functional traits, dysfunctional traits tend to be more manageable and modifiable when referred to a registry age younger than age 14. The structure always remains the same, with yes/no responses, but the time reference is extended to the last quarter (as is the case in PICI‐TA‐3), administered by specialized staff, in support of the patient. On the subject of data interpretation, the PICI‐C‐3 modifies the assignments of some items and their specific dysfunctional trait activation values, avoiding more errors of symptomatological or diagnostic overlap, in the clinical interview; for the same reason, the assignment codes for the absorbing (able to absorb) and absorbed (able to be absorbed) categories have also been revised. Finally, it is officially established that the diagnosis in PICI‐C‐3 is both structural (relating to the category most representative of dysfunctional traits) and functional (relating to the representative categories immediately following the first); in this sense, the first category represents the dysfunctional structure of reference, while the subsequent categories define the subject's pathological functioning, about the identified structure. The category inhibited from uninhibited is definitively separated, in attachment, thus becoming 19 categories and no longer 18 (previously, category No. 13 was separated into “13a” and “13b,” as was the case for narcissism in PICI‐TA‐3).
c) Completion of either section A or section B depends on registry age, and one automatically excludes completion of the other.
d) The concluding diagnosis of dysfunctionality, for both PICI‐TA‐3 and PICI‐C‐3, is structured based on the highest score of dysfunctional traits found, per single category (25 for PICI‐TA‐3 and 19 for PICI‐C‐3).
e) The concept of egosyntonic personality disorders is mitigated by the model underlying the PICI, and therefore the possible presence of a patient's egosyntonicity concerning his or her symptoms is discernible only when the psychopathological impairment is severe (>7/9 dysfunctional traits), while in all other cases, it is possible to witness egodystonic patients, concerning their symptoms, while still having values relevant to the diagnosis of personality disorder.
f) The neurotic area of disorders includes six specificities, but they should be treated as if they were a unitary disorder^25^ (referred to as “neurotic personality disorder”).
g) The complexity of the psychopathological diagnosis of borderline and bipolar disorder must be addressed during therapy and mediated by the responses obtained during psychotherapy, as manic and depressive traits induce the activation of both bipolar and borderline traits, as they are common. Indeed: in the bipolar patient, there is a marked tendency toward emotional and mood instability, which is more rigid than that of the borderline and is enriched by irrational beliefs taken to extremes, between depressive and manic episodes representing the patient's two main modes (albeit with four different possible sub‐hypotheses), however, depressive episodes are common to depressive disorder, while manic episodes are common to borderline disorder and some psychotic activations; in the borderline patient, there is a greater and more pronounced tendency toward impulsivity and aggression, with more emotional and affective fluctuations (as it is a relationship disorder), sudden and rapid, and depressive and manic episodes that are shorter and more circumscribed in the relevant timeframe than in the bipolar. It is, therefore, more than correct to separate the two interpretative hypotheses, precisely because of their peculiar characteristics of the behavioral and emotional‐affective framework, although they have several commonalities that would appear to overlap. Therefore, modifications have been made to the absorbing patterns of the categories and the activation codes of the dysfunctional traits, precisely to facilitate the most correct interpretation possible.
h) In PICI‐TA‐3, the sadistic‐masochistic and narcissistic (overt‐covert) scales seem to influence each other, and therefore during the clinical interview, it is also useful to subject the patient to psychometric tests assessing individual sexual matrix^26^ and sex‐related profiles,^27^, ^28^, ^29^, ^30^, ^31^, ^32^, ^33^, ^34^, ^35^, ^36^, ^37^, ^38^, ^39^, ^40^, ^41^, ^42^, ^43^, ^44^, ^45^, ^46^, ^47^, ^48^, ^49^, ^50^, ^51^, ^52^, ^53^, ^54^, ^55^, ^56^, ^57^, ^58^, ^59^, ^60^, ^61^, ^62^, ^63^, ^64^, ^65^, ^66^, ^67^, ^68^, ^69^, ^70^, ^71^, ^72^, ^73^, ^74^, ^75^, ^76^, ^77^, ^78^, ^79^, ^80^, ^81^, ^82^, ^83^, ^84^, ^85^, ^86^, ^87^, ^88^, ^89^, ^90^, ^91^, ^92^ to ascertain whether these activations are more consistently related to paraphiliac disorder (common secondary) or personality structure.
i) The identification of common secondary disorders (PICI‐SD‐3, section C) must be reported in the patient's history and technical file and must be corroborated by specialized instrumental and testistic investigations; in the absence of such documentation, the therapist must request its implementation and only later close the file with the reference to the disorder possibly identified.
j) The completion of the functional personality traits part (PICI‐FT‐3, section D) involves the assignment of three distinct scores: (a) a partial score from 0 to 5, per individual behavior style; (b) a final score referring to the area of functioning (24 areas) from 0 to 25; (c) a final score referring to the personality type (6 types) from 0 to 130 for the emotional one, from 0 to 120 for the dynamic one, from 0 to 110 for the rational and phlegmatic one, from 0 to 85 for the reflective one, and from 0 to 45 for the instinctive one. Concerning areas of functioning, scores are classified according to five areas (red = poor functioning, orange = medium functioning, yellow = sufficient functioning, green = good functioning, purple = excellent functioning), as well as for personality types (Supporting information 11–14).

The method used consists of two consecutive operations: the first is related to the clinical interview, based on anamnestic and documentary narrative evidence, with an interview related to the emotional and perceptual‐reactive experience of the patient, according to the PHE Model,^4^ updated to the new version PHEM‐2^37^; the second is related to the administration in the first instance of the Perrotta Integrative Clinical Interviews‐3 (PICI‐3) and the Minnesota Multiphasic Personality Inventory‐2‐Restructured Form (MMPI‐2‐RF)^93^ relative to Section A outcomes, and the Child Behavior Checklist (CBCL)^94^ relative to Section B outcomes. The comparisons made served to give greater robustness to the conclusions, although Sections A (PICI‐TA‐3) and B (PICI‐C‐3) are constructed based on the symptoms described by the DSM‐5‐TR,^17^ and thus perfectly fit. In contrast, the outcomes related to Section C (PICI‐SD‐3) are derived from the clinical records produced by the patients and therefore do not need comparison for validation. In contrast, the outcomes related to Section D (PICI‐FT‐3) were compared with the outcomes of the Big Five Personality Test (Big5).^95^ In the second instance, after 1 month, for validation of the stability of the test, PICI‐3 was reused, to enable a comprehensive statistical analysis for validation of the latter by comparing the data with the first administration. For administration, a protected link was used to guarantee anonymity, official translations of the psychometric tests used, and a private IT management program in Excel format for the data obtained, obtained from entering the answers on the protected link. The time required for the complete administration of the PICI‐3 is estimated at 45–60 min, excluding the integrated clinical interview of approximately 30–60 min. The research steps were divided as follows: 1. Selection of the population sample, according to the parameters given in the next Section 2. Clinical interview with each population group, as indicated in the next paragraph. 3. Administration of psychometric tests. 4. Data processing after administration. 5. Comparison of the data obtained. The statistical methods used are descriptive comparison, comparison of averages, and t‐test, using IBM SPSS Statistics software.

### 2.3 Setting and participants

Inclusive criteria for the selection of the population are: (1) Age between 8 years and 90 years; (2) Italian nationality; (3) absence of neurodegenerative disorders or severe genetic diseases capable of impairing cognitive functioning. Exclusive criteria for the selection of the population are: (1) Age ≤7 years and ≥91 years; (2) foreign nationality (all individuals who did not have Italian nationality by birth under national law) and with one or no parents of Italian nationality by birth; (3) presence of neurodegenerative disorders or severe genetic diseases capable of impairing cognitive functioning. The chosen setting, tender standing during the protracted pandemic period (already in progress since the beginning of the present research), is the online platform via Skype and WhatsApp Video Calls, both for clinical interviews and administration. PICI‐3 was administered in Italian language. The participation of individuals under the age of 18 has been expressly authorized by both parents or in their absence by the legal representative. The present research work was carried out from September 2021 to September 2023. All participants were guaranteed anonymity and the ethical requirements of the Declaration of Helsinki were met. The research is not funded by anyone, and it is free of conflicts of interest.

## 3 RESULTS

### 3.1 Development and regulation of the questionnaire (PICI‐3)

The PICI‐3 identifies the subject's functional and dysfunctional personality traits by measuring the degree of clinical impairment about the symptomatology described in the DSM‐5‐TR and the PICI's basic model (IPM), described in the introduction of this research article. It refers to the developmental stages of youth (8–10 years), preadolescence (11–13 years), adolescence (14–18 years), adulthood (19–69 years), and old age (70–90 years). Several renovations have been made compared to the previous version, both about structural and functional elements, as indicated in the materials and methods section of this article, and the intent is to obtain a realistic snapshot of the subject's mental health status, keeping in mind that the assessment must always be updated based on clinical findings. Section A consists of 173 items, Section B of 128 items, Section C of 12 items, and finally the last Section D consists of 24 items. The final assessment of the results takes into account both the structural elements that depend on innate tendencies, childhood experience, and the socio‐familial and environmental context, especially in the first 12–14 years of life and the functional elements that depend on adolescent and adult experience, with behavioral reinforcers, education received, and lived life experience.

### 3.2 Court study

**Figure 2:**
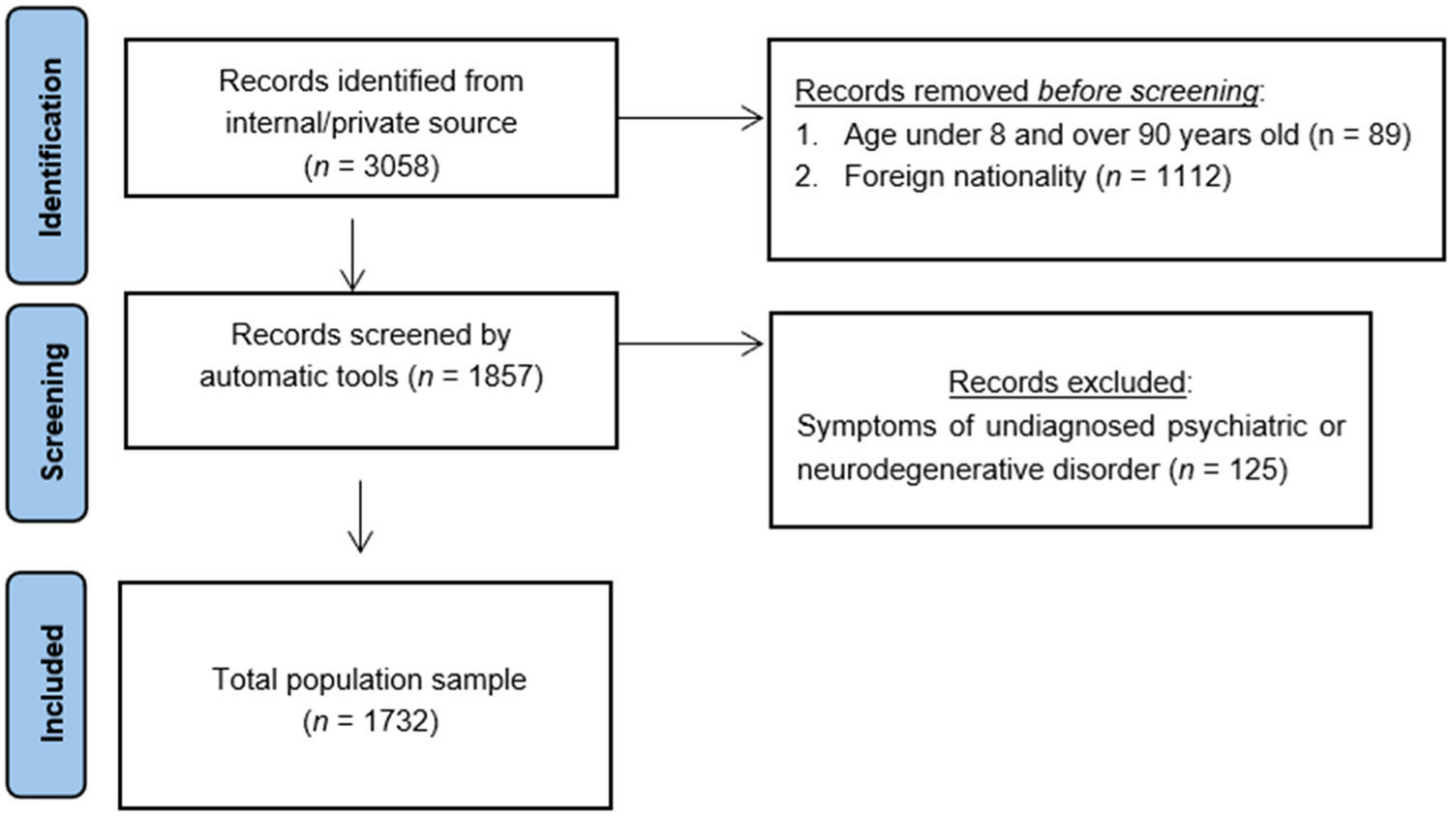
Flowchart of the population sample selection process. [Color figure can be viewed at wileyonlinelibrary.com]

The sample of the selected population is 1732 participants (712/m; 1020/f) for the entire study. The drop‐out rate is 0/1732 (0%). The least populous age groups are the most extreme, the youngest (8–13 years old) and the most mature (70–79 years old and 80–90 years old), with an equal distribution in the middle age groups. The total numerosity is considered representative, of the stated aims and objectives (Table 4). There were 1732 patients included, while those excluded from the study were 1326 (Figure 2).

**Table 4:** Population sample (numerousness): Descriptive statistical analysis.

Age	Male	Female	Total
8–13	30 (4.2%)	70 (6.9%)	100 (5.8%)
14–18	76 (10.7%)	116 (11.4%)	192 (11.1%)
19–29	108 (15.2%)	125 (12.3%)	233 (13.5%)
30–39	109 (15.3%)	136 (13.3%)	245 (14.1%)
40–49	119 (16.8%)	143 (14.0%)	262 (15.2%)
50–59	99 (13.9%)	132 (12.9%)	231 (13.3%)
60–69	90 (12.6%)	139 (13.6%)	229 (13.2%)
70–79	65 (9.1%)	95 (9.3%)	160 (9.2%)
80–90	16 (2.2%)	64 (6.3%)	80 (4.6%)
Total	712 (41.1%)	1020 (58.9%)	1732 (100%)

### 3.3 Validation of the questionnaire (PICI‐3)

Structurally, the PICI‐3 consists of four different sections (A, B, C, D), each with its precise scale of interpretation, as stated in the methods and materials section of this research article. For this reason, the comparison for validation purposes was made (Table 5).

**Table 5:** Comparison of psychometric tests (meads), for validation.

Test to be validated	Test used for validation
PICI‐TA‐3 (Section A)	Minnesota Multiphasic Personality Inventory‐2—Restructured Form (MMPI‐2‐RF)^93^
PICI‐C‐3 (Section B)	Child Behavior Checklist (CBCL)^94^
PICI‐DS‐3 (Section C)	Hospital clinical documentation
PICI‐FT‐3 (Section D)	Big Five Personality Test (Big5)^95^

The PICI‐TA‐3 consists of 173 items and can detect based on symptomatology suffered dysfunctional traits based on 25 different personality disorders; the test chosen for validation comparison, which comes closest to the results of the test, is the Minnesota Multiphasic Personality Inventory‐2‐Restructured Form (MMPI‐2‐RF),^93^ although it is possible to compare in the former case the diagnostic outcome of the specific disorders based on the activations of the individual dysfunctional personality traits (0–9 traits per disorder), while in the latter case, it is possible to derive from the activated scales the symptomatology incorporating the personality disorders identified by the PICI‐TA‐3. The summation of individual dysfunctional traits defines the psychopathological diagnosis. The MMPI‐2‐RF consists of 338 items, with double response alternatives (true/false), which go to construct 51 scales of which nine are validity, three superordinated (H‐O), nine restructured clinical (RC), 23 specific problem scales (SP), two interest scales, and five personality pathology‐related scales (PSY‐5); the H‐O, RC, interest, SP, and PSY‐5 scales are referred to as the “substantive scales.” Comparing symptomatological outcomes with 40/51 scales, validity scales and scales of interest having been excluded, and normalizing the results to obtain direct data comparison, the statistical analysis confirmed 99.3% data compatibility, Pearson's coefficient (*R*) of 0.999 and *p* < 0.001.

The PICI‐C‐3 consists of 128 items and can detect based on symptomatology suffered dysfunctional traits based on 19 different disorders (not personality, as before the age of 14 years it is not possible); the test chosen for validation comparison, which comes closest to the results of the test, is Child Behavior Checklist (CBCL),^94^ although it is possible to compare in the former case the diagnostic outcome of the specific disorders based on the activations of individual dysfunctional personality traits (0–9 traits per disorder), while in the latter case, it is possible to derive from the lists of dysfunctional behaviors the symptomatology that is incorporated and counted among the symptoms of the disorders identified by the PICI‐C‐3, also because of studies on attachment and childhood trauma.^96^, ^97^ The CBCL consists of 128 items between the first and second parts (social skills with behavior and emotional problems). Comparing the symptom outcomes with the outcomes from the administration, and normalized the results to obtain the direct comparison of data, statistical analysis confirmed 94.1% data compatibility, Pearson's coefficient (*R*) of 0.969 and *p* < 0.001.

The PICI‐DS‐3 consists of 12 items and can detect based on the symptomatology suffered the disorders that the PICI model considers secondary common to the primary diagnosis (structural and functional). As they are considered only if a clinical hospital record is produced, it was not deemed necessary to proceed to validation, being a simple named list to complete the subject's medical history form.

The PICI‐FT‐3 consists of 24 items and can detect individual functional personality traits of the subject; in particular, it can detect both the preponderant personality type and behavioral styles; the test chosen for validation comparison, which comes closest to the results of the test, is the Big Five Personality Test (Big5),^95^ which again can only be compared with the results of individual areas and not the outcome, due to obvious structural divergences between the two tests. The BIG5 consists of 120 items identifying characteristics of five different domains: openness to experience, extroversion, conscientiousness, agreeableness, and neurosis. Comparing the functional outcomes with the two internal subsections, and normalized the results to obtain direct data comparison, statistical analysis confirmed 89.4% data compatibility, Pearson's coefficient (*R*) of 0.797 and *p* < 0.001.

**Figure 3‐5:**
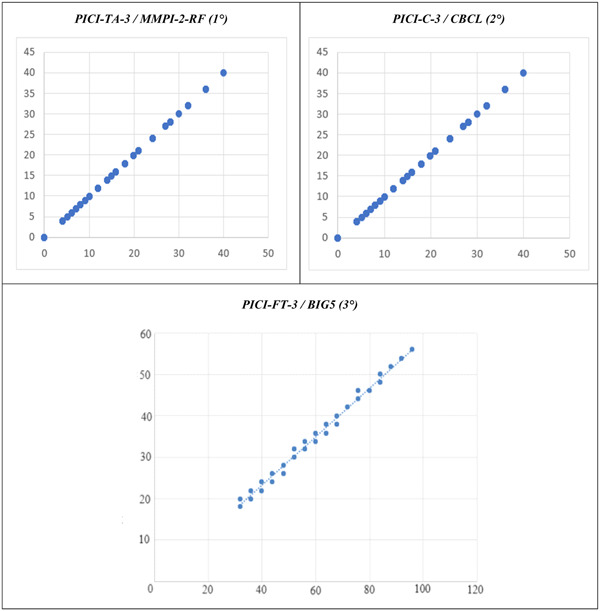
Comparison of the tests: PICI‐TA‐3/MMPI‐2‐RF (1°); PICI‐C‐3/CBCL (2°); PICI‐FT‐3/BIG5 (3°). Perrotta Integrative Clinical Interviews 3 (PICI‐3); Minnesota Multiphasic Personality Inventory‐2—Restructured Form (MMPI‐2‐RF); Child Behavior Checklist (CBCL); Big Five Personality Test (Big5). *t*‐Test for paired data. [Color figure can be viewed at wileyonlinelibrary.com]

The results of the three comparisons are shown in the following graph (Figures 3–5).

#### 3.3.1 Coefficient of stability

A binary correlation analysis was conducted between the first administration of the PICI‐3 and the second administration, which occurred after 1 months (with the drop‐out of the 0.0%), to check the stability of the test (and in the absence of specific psychotherapeutic intervention), obtaining a Pearson's coefficient (*R*) of 0.999, with *p* < 0.001 (Table 6).

**Table 6:** Statistical analysis for the evaluation of the stability coefficient, expressed by the Pearson.

	N_population	Mean (*M*)	Standard deviation (SD)
PICI‐3 (first)	1732	63.6	28.1
PICI‐3 (1 month)	1732	63.5	28.1

	**N_population**	**Correlation**	**Significance (*p*)**
PICI‐3 (first) <‐> PICI‐3 (1 month)	1732	0.999	<0.001

## 4 DISCUSSION

The PICI‐3, used to identify the functional and dysfunctional traits of the subject's personality by measuring the degree of clinical impairment about the symptomatology described in the DSM‐5‐TR and based on the PICI (IPM) model is the world's first psychometric instrument capable of taking a snapshot of both structural and functional elements of the whole personality. For this reason, comparison with other psychometric instruments is extremely complex because of obvious dissimilarities in the basic model and structure. Although the first three sections are checklists based on the DSM‐5‐TR, however, validation was carried out by comparison with tests that are closest in structure and function. Based on the proposed model, the PICI‐3 confirms itself as more innovative than the previous versions, especially in terms of psychopathological diagnosis and in identifying the structure and functioning of functional personality traits, identifying both the predominant personality type (out of six possible choices) and the individual areas that characterize the sum of the activated traits. The statistical analysis thus confirmed that the PICI‐3, in all its sections, has a well‐defined and stable construct, with the variables well‐represented and positively correlated with the selected psychometric constructs.

## 5 LIMITATIONS, IMPLICATIONS FOR CLINICAL PRACTICE, AND PROSPECTS

In this validation analysis, the main limitation found concerns the co‐items, which cannot be compared with the entirety of the selected psychometric tools, not even with the final total score (except in the case of normalization), as the basis are different and identify items that are comparable through the partial outcome but not their total; this limitation, however, did not prevent the statistical analysis carried out from giving good results in terms of stability, effectiveness, and efficiency, thus validating the PICI‐3 psychometric instrument. Through the use of the underlying model, it was therefore possible to update, modify, and renew a multi‐sectoral questionnaire which concretizes the need to investigate, in terms of functioning/dysfunction, the capacity of personality traits. The perspectives will be directed toward the administration of the PICI‐3 to a wider population, especially in the younger and more mature generational categories, less represented in this study, to refine the evaluation in the diagnostic phase with emphasis on clinical psychopathological correlations.

## 6 CONCLUSIONS

This work discusses the development and validation of Perrotta Integrative Clinical Interviews‐3 (PICI‐3). PICI‐3 is a psychometric tool used to identify functional and dysfunctional personality traits in different age groups and to diagnose psychopathological disorders. PICI‐3 is divided into four sections that focus on dysfunctional features in children, adolescents, adults, and older adults, as well as common secondary disorders and functional features. It is found that PICI‐3 is a reliable and effective diagnostic tool for psychopathological disorders, which has certain clinical significance. PICI‐3 is a psychometric tool with a well‐defined and stable construct, with the variables well represented and positively correlated with another already validated construct, to identify the functioning or dysfunction of personality traits.

## AUTHOR CONTRIBUTIONS

Giulio Perrotta conceptualized this study, collected and analyzed all data, finalized and approved it.

## CONFLICT OF INTEREST STATEMENT

The author declares no conflict of interest.

## ETHICS STATEMENT

All participants were assured of compliance with the ethical requirements of the Charter of Human Rights, the Declaration of Helsinki in its most up‐to‐date version, the Oviedo Convention, the guidelines of the National Bioethics Committee, the standards of “Good Clinical Practice” (GCP) in the most recent version, the national and international codes of ethics of reference, as well as the fundamental principles of state law and international laws according to the updated guidelines on observation studies and clinical trial studies. According to Legislative Decree No. 52/2019 and Law No. 3/2018, this research does not require the prior opinion of an Ethics Committee in the implementation of Regulation (EU) no. 536/2014. In compliance with Regulation (EU) 2017/745, the Declaration of Helsinki, and the Oviedo Convention, the scientific research contained in the manuscript: (a) does not involve new or already on‐the‐market drugs or medical devices; (b) does not involve the administration of a new or already on‐the‐market drug or medical device; (c) is not for commercial purposes; (d) is not sponsored or funded; (e) the participants have signed the informed consent and data processing, in compliance with applicable national and EU regulations (f) refers to non‐interventional but observational‐comparative diagnostic topics, for validation of the newly proposed questionnaire; (g) the population sample was collected at a date before the start of this study and is part of a private, non‐public database. I remain at your disposal.

## Supporting information

Supporting information.

## Data Availability

The subjects who participated in the study requested and obtained that GP be the sole examiner during the therapeutic sessions, and that the participant's data is in an exclusively anonymous form. The data sets generated and/or analyzed during the current study are available from the corresponding author upon reasonable request.
