# Supplementary material for: Perrotta Integrative Clinical Interviews‐3 (PICI‐3): Development, regulation, updation, and validation of the psychometric instrument for the identification of functional and dysfunctional personality traits and diagnosis of psychopathological disorders, for children (8–10 years), preadolescents (11–13 years), adolescents (14–18 years), adults (19–69 years), and elders (70–90 years)
Source: Ibrain. 2024 Feb 13;10(2):146–63. doi: 10.1002/ibra.12148 (PMC11193867; doi:10.1002/ibra.12148)
Supplement: Supplementary file 1 — Supporting information. [file IBRA-10-146-s001.docx]

**Supporting information 1**: Structural categories of the dysfunctional personality of Perrotta Integrative Clinical Interviews (adolescent and adult) - PICI-1TA, with their respective characteristics

| ***Personality disorder*** | **N.** | **Definition and typologies** | **Dysfunctional area** |
| --- | --- | --- | --- |
| *Anxious personality disorder* | 1 | It is a habitual, stable, persistent and pervasive pattern, with onset in childhood but evolves structurally in adolescence, characterized by a perceived dysfunctional state of anxiety, low anxiety tolerance and high vulnerability to frustration:   - 1. perceived dysfunctional state of anxiety;   2. the rigidity of thinking;   3. complaining and/or ruminating;   4. fixed or obsessive thoughts related to the anxious state;   5. easy irritability and/or fatigability;   6. low tolerance for anxiety and/or frustration;   7. total or partial inability to perform normal daily activities;   8. marked episodes of anxiety that result in panic and/or striking hysterical symptoms;   9. psychomotor agitation, with restlessness, muscle tension and/or difficulty in finding concentration.   An anxious episode alone for a specific event, without chronic and persistent symptoms, is not sufficient for the diagnosis of anxious personality disorder but will have to be defined as an "*anxious episode*". If, however, the anxious episodes follow one another, with or without specific events, for at least one month, one will have to speak of "*multiple complex anxious episodes*"; if they last for more than six months, one will finally have to speak of "*generalized type of anxious personality disorder*".  When the anxiety focuses on the social context, giving rise to a free anxiety phenomenon without phobic symptoms, one will have to speak of "*anxious personality disorder of the social type*".  When the anxious state manifests with deep anxiety, fear of death and striking somatic symptoms (e.g., chest tightness, sweating, shortness of breath, flushing and tingling), one will have to speak of "*panic-type anxious personality disorder*".  When the anxious state is manifested by intense fear and feelings of helplessness or horror, recurrent intrusive unpleasant memories (images, thoughts, or perceptions, nightmares, and unpleasant dreams, acting or feeling as if the traumatic event were recurring, intense psychological distress at exposure to internal or external triggers that symbolize or resemble some aspect of the traumatic event, physiological reactivity or exposure to internal or external triggers that symbolize or resemble some aspect of the traumatic event persistent avoidance of stimuli associated with the trauma and dampening of general reactivity, difficulty falling asleep or maintaining sleep, irritability or angry outbursts, difficulty concentrating, hypervigilance and exaggerated alarm responses), following a traumatic event, one will have to speak of "*post-traumatic stress disorder*", which if not effectively reprocessed could develop first into "*adjustment disorder*" (as codified by the DSM-V-TR) and then into "*post-traumatic personality type anxiety disorder*". | Neurotic domain (Cluster A) |
| *Phobic personality disorder* | 2 | It is a habitual, stable, persistent and pervasive pattern, with onset in childhood but evolves structurally in adolescence, characterized by phobic manifestations not justified by the possible source of danger, rigidity of thought and avoidance:  2.1. phobic manifestations not justified by the possible source of  danger;  2.2. the rigidity of thought;  2.3. avoidance of the possible source of danger;  2.4. fixation and/or obsession;  2.5. chronicity of the phobia and/or multiplicity of manifestations on  multiple phobic objects;  2.6. total or partial inability to perform normal daily activities;  2.7. low tolerance for anxiety and/or frustration;  2.8. marked discomfort experienced in potentially non-threatening or  stressful situations;  2.9. marked episodes of anxiety that result in panic and/or striking  hysterical symptoms.  Phobia for a specific object alone (e.g., spiders), with chronic and/or obsessive symptoms, is not sufficient for the diagnosis of phobic personality disorder but will have to be defined as "*single specific phobia*"; if the sources are multiple, it will be referred to as "*multiple specific phobias*".  When the phobia focuses on the social context, giving rise to an anxiety-free phenomenon, one will have to speak of "*social phobic personality disorder*". | Neurotic domain (Cluster A) |
| *Avoidant personality disorder* | 3 | It is a habitual, stable, persistent, and pervasive pattern, with onset in childhood but evolves structurally in adolescence, characterized by excessive fear to the point of paranoia, avoidance (but resulting in suffering from social isolation), and low self-esteem:  3.1. excessive and/or unfounded fear;  3.2. avoidance of potentially stressful circumstances and/or attempts at  avoidance;  3.3. delegation of responsibility;  3.4. low self-esteem;  3.5. unwillingness to be involved in activities communal and/or  collective;  3.6. marked anxiety when activities become common and/or collective;  3.7. marked concern about judgment, criticism, and rejection from  people;  3.8. reluctance to take risks and dangers, even calculable ones;  3.9. fear of derision and/or humiliation for one's mistakes. | Neurotic domain (Cluster A) |
| *Obsessive personality disorder* | 4 | It is a habitual, stable, persistent and pervasive pattern, with onset in childhood but evolves structurally in adolescence, characterized by obsessions, mental rigidity and need for control:  4.1. obsessions;  4.2. compulsions;  4.3. perfectionism;  4.4. mental rigidity;  4.5. need for control;  4.6. marked discomfort in public;  4.7. concern about one's own and/or others' health status, without  justifiable reasons;  4.8. altered perceptual state, without delusions or hallucinations, about  one's own or another's body;  4.9. delusional and/or paranoid thoughts and/or beliefs.  When the obsession is without compulsion, it will be referred to as "*simple obsessive personality disorder*"; on the other hand, if there are multiple obsessions but still without compulsions, it will be referred to as "*complex obsessive personality disorder*". If obsessions and compulsions exist simultaneously, it will be called "*obsessive-compulsive personality disorder*".  When the obsession concerns aesthetic appearance one will have to speak of "*obsessive personality disorder of the body dysmorphic type*".  When the obsession concerns accumulation one will have to speak of "*obsessive personality disorder of the accumulative type*".  When the obsession concerns setting fires one will have to speak of "*obsessive personality disorder of the pyromaniac type*", unless otherwise attributed psychopathologically (e.g., pyromania as a symptom of antisociality or psychopathy).  When the obsession is about stealing objects, one should speak of "*obsessive personality disorder of the kleptomanic type*".  When the obsession is about pulling out hair or causing excoriations to oneself, one will have to speak of "*obsessive personality disorder of the injury type*". | Neurotic domain (Cluster A) |
| *Somatic personality disorder* | 5 | It is a habitual, stable, persistent, and pervasive pattern, with onset in childhood but evolves structurally in adolescence, characterized by somatic symptoms in the absence of relevant clinical data, preoccupation with health status, and low frustration tolerance:  5.1. somatic symptoms in the absence of relevant clinical data;  5.2. concern about health status;  5.3. concern about one or more illnesses;  5.4. low tolerance for anxiety and frustration;  5.5. seeking answers outside the health care setting, despite doctors'  advice;  5.6. difficulty concentrating and fulfilling one's tasks and duties;  5.7. obsessive and/or paranoid thinking;  5.8. complaining and ruminating about the state of health or symptom;  5.9. low self-esteem and/or insecurity. | Neurotic domain (Cluster A) |
| *Manic personality disorder* | 6 | It is a habitual, stable, persistent, and pervasive pattern, with onset between the ages of 5 and 10 years but evolves structurally into adolescence, characterized by dysfunctional alteration of mood tone, flight of ideas, and psychomotor agitation:  6.1. dysfunctional alteration of mood tone;  6.2. flight of ideas;  6.3. psychomotor agitation;  6.4. prodigality and/or excessive spending;  6.5. flight of ideas and/or increased speed of ideas, whether or not  involving forgetfulness and/or activities left unfinished while  starting new ones;  6.6. increased libido and/or sociability and/or the need to stay at home  Or in the office postponing appointments to work on the idea;  6.7. ideas of grandiosity and/or increased self-esteem;  6.8. tendency to delusional episodes;  6.9. hyperarousal and/or hyperactivity, with or without logorrhea  and/or sudden changes in thought flows.  There are two forms of this disorder:  1) *Type I*: the form described above;  2) *Type II*: in the absence of delusional tendency and modest  hyperactivation, the episodes are hypomanic.  The co-presence of depressive or dysthymic symptoms and maniacality or hypomaniacality configures the diagnosis of "*Bipolar personality disorder*". | Neurotic domain (Cluster A) |
| *Bipolar personality disorder* | 7 | It is a habitual, stable, persistent, and pervasive pattern, with onset between the ages of 5 and 10 years but structurally evolving into adolescence, characterized by sudden mood fluctuations, manic and/or depressive states, and/or abrupt alternate and emotional instability:  7.1. sudden fluctuations in mood;  7.2. emotional instability;  7.3. relational and/or social instability;  7.4. manic, depressive and/or mixed episodes;  7.5. tendency to active and/or passive manipulation;  7.6. low tolerance to frustration and anxiety;  7.7. tendency to irritability;  7.8. low tolerance for criticism;  7.9. mood tending to be dysphoric (with or without unpleasant feelings,  frustration, pessimism, tension, irritability, anxiety, and  psychomotor agitation).  There are four main forms:  a) *Type I bipolarity*: overt alternation of manic episodes with  depressive episodes;  b) *Type II bipolarity*: alternating hypomanic episodes with depressive  or dysthymic episodes;  c) *Type III bipolarity*: prevalence of the depressive or manic state, with  a tendency to fluctuating dysthymic or hypomanic episodes.  d) *Type IV (or cyclothymic) bipolarity*: alternating hypomanic and  dysthymic episodes. | Latent domain (Cluster B) |
| *Emotional-behavioural personality disorder* | 8 | It is a habitual, stable, persistent, and pervasive pattern, with onset around age 5 but evolving structurally in adolescence, characterized by the systematic and persistent violation of social norms and civil commonality (not necessarily in violation of the law), negative consequences resulting from behaviours, and dysfunctional management of one's basic emotions:  8.1. systematic and persistent violation of social norms and/or civil  commonality;  8.2. negative consequences arising from behaviours;  8.3. dysfunctional management of one's basic emotions;  8.4. low tolerance for anxiety and/or frustration;  8.5. episodes of explosive and/or uncontrolled or otherwise unjustified  anger concerning the event, later compensated with guilt,  shame or remorse;  8.6. impulsiveness and/or tendency for active manipulation;  8.7. recklessness and/or excessive instinctiveness;  8.8. verbal and/or physical aggression to objects, people and/or  animals;  8.9. violation of rules and/or regulatory dictates, relevant to national  law.  The symptoms suffered must not meet the requirements of *antisocial personality disorder*. | Latent domain (Cluster B) |
| *Dependent personality disorder* | 9 | It is a habitual, stable, persistent, and pervasive pattern, with onset around age 4 but evolving structurally into adolescence and adulthood, characterized by strong insecurity, tendency to need approval, and delegation of responsibility:  9.1. strong insecurity and/or tendency for passive manipulation;  9.2. tendency to need approval from others;  9.3. delegation of responsibility;  9.4. difficulty in making daily decisions;  9.5. inclinations to strive for the benefit and support of others;  9.6. feelings of discomfort and/or helplessness when alone without  asking for help or advice;  9.7. unrealistic and excessive worries;  9.8. fear of being abandoned and having to take care of themselves  alone;  9.9. low self-esteem. | Latent domain (Cluster B) |
| *Depressive personality disorder* | 10 | It is a habitual, stable, persistent, and pervasive pattern, with onset around age 6 but evolving structurally into adolescence and adulthood, characterized by depressed mood, low self-esteem, and a marked decrease in interests and pleasures:  10.1. depressed mood;  10.2. low self-esteem and/or tendency to passive manipulation;  10.3. a marked decrease in pleasure in performing interests and  Activities and/or tendency to boredom;  10.4. significant weight gain or decrease;  10.5. agitation and/or psychomotor slowdown;  10.6. lack of energy and/or easy fatigue;  10.7. marked feelings of self-evaluation, inappropriateness and/or  guilt;  10.8. reduced ability to concentrate on activities;  10.9. recurring negative or melancholic and/or death-related thoughts  that are not provoked by actual events (e.g., bereavement).  The "bereavement" event can trigger a depressive tendency, even to the point of arising in "*persistent bereavement depressive personality disorder*".  When the suffered symptomatology allows one to carry out one's work or activities, albeit maintaining the behavioural and mood characteristics of the depressed patient, one will have to speak of the attenuated form of "*dysthymic-type depressive personality disorder*". If the depressive manifestation is caused by the birth of a child and persists beyond one month, one will have to speak of "*acute depressive personality disorder of the postpartum type*", while if it exceeds six months, one will have to speak of "*chronic depressive personality disorder of the postpartum type*" (in which symptoms of psychotic activation may also be present). | Latent domain (Cluster B) |
| *Borderline personality disorder* | 11 | It is a habitual, stable, persistent, and pervasive pattern with onset around age 8 but evolves structurally into adolescence and adulthood characterized by emotional instability, sudden mood swings, and impulsivity:  11.1. emotional instability and/or impulsivity in interpersonal  relationships;  11.2. sudden mood swings;  11.3. active and/or passive manipulative tendency;  11.4. desperate efforts to avoid abandonment (real and/or imagined);  11.5. dysfunctional and/or unstable self-image;  11.6. marked impulsivity capable of harming them;  11.7. persistent feelings of emptiness;  11.8. sudden anger and unwarranted aggression;  11.9. irrational thoughts and beliefs, resulting in all or part of the  psychotic sphere. | Latent domain (Cluster B) |
| *Histrionic personality disorder* | 12 | It is a habitual, stable, persistent, and pervasive pattern, with onset around age 8 but evolving structurally into adolescence and adulthood characterized by the immoderate need for attention, fear of abandonment (real or presumed), theatricality and drama of actions:  12.1. inordinate need for attention;  12.2. discomfort when they are not the centre of attention;  12.3. real and/or presumed fear of abandonment;  12.4. theatricality and drama of their self;  12.5. high suggestibility;  12.6. vague and/or impressionistic language;  12.7. changeable personal and/or relational instability;  12.8. constant use of physical appearance to attract attention, including  through more or less explicit sexual conduct;  12.9. manipulative, provocative and/or seductive mode of expression. | Latent domain (Cluster B) |
| *Narcissistic personality disorder, OVERT type* | 13 | It is a habitual, stable, persistent, and pervasive pattern, with onset around age 4 but evolving structurally into adolescence and adulthood, characterized in the "overt" form by poor empathy, the idea of grandiosity, and excessive self-esteem:  13.1. poor or absent empathy;  13.2. wholly or partially unfounded beliefs of being unique and special  and/or ideas of grandiosity;  13.3. excessive self-esteem and/or arrogance;  13.4. irrational beliefs of being envied by others for his or her position  and/or inherent human, personal and/or moral qualities;  13.5. concerns related to fantasies of success and/or perfection;  13.6. need for admiration;  13.7. irrational belief that he/she deserves what he/she desires and/or  dreams and/or aspires for;  13.8. manipulative exploitation of people and/or circumstances for  one's gain, whether or not using guilt, shame,  personal relationships, professional activity, and/or sex;  13.9. use of physical, verbal and/or psychological violence, with or  without aggression. | Latent domain (Cluster B) |
| *Narcissistic personality disorder, COVERT type* | 14 | It is a habitual, stable, persistent, and pervasive pattern, with onset around age 4 but evolving structurally into adolescence and adulthood, characterized in the "covert" form by low self-esteem, intolerance of criticism and judgment, complaining, and passive-aggressive behaviour:  14.1. low self-esteem, aimed at attracting attention;  14.2. low tolerance to criticism and/or judgments;  14.3. use of complaints and/or whining to get attention;  14.4. passive-aggressive conduct;  14.5. exaggerated underestimation;  14.6. irrational fixations and/or beliefs;  14.7. striking somatic and/or hysterical symptoms;  14.8. lack of empathy and/or little or no sensitivity concerning the  needs of others;  14.9. excessive need for control. | Latent domain (Cluster B) |
| *Antisocial personality disorder* | 15 | It is a habitual, stable, persistent, and pervasive pattern, with onset around age 8 but evolving structurally in adolescence and adulthood characterized by a lack of empathy, lack of remorse, and disregard for rules and social roles:  15.1. narcissistic tendencies;  15.2. lack of empathy;  15.3. lack of remorse, feelings of guilt and/or shame;  15.4. lack of respect for rules and social roles;  15.5. marked tendency to delinquency and/or active manipulation,  even without a criminal record or judicial problems;  15.6. tendency to aggressiveness and/or provocation;  15.7. low tolerance for frustration and/or anxiety;  15.8. prevalence of negative feelings;  15.9. tendency to impulsiveness and/or irresponsibility. | Latent domain (Cluster B) |
| *Sadistic personality disorder* | 16 | It is a habitual, stable, persistent, and pervasive pattern, with onset around age 8 but evolving structurally in adolescence and adulthood characterized by taking pleasure from others' suffering (outside the sexual sphere), manipulation of people and/or situations for personal vantage (to the detriment of others), and prevalence of negative feelings:  16.1. enjoyment from the suffering of others, outside the sexual sphere; 16.2. manipulation of people and circumstances for personal  advantage, to the detriment of other people;  16.3. prevalence of negative feelings;  16.4. discomfort in the presence of pleasant events and positive  feelings;  16.5. need to make people suffer, humiliate and inflict pain, to  gain pleasure from it;  16.6. the pathogenic belief that one has the right to make others suffer;  16.7. unconscious abuse of primitive defense mechanisms;  16.8. narcissistic tendencies;  16.9. emotional and/or situational reversal of pleasure/pain.  If the symptomatology is alternated with the masochistic pattern, with a greater or lesser prevalence, one must speak of "*sadomasochistic personality disorder*". | Latent domain (Cluster B) |
| *Masochistic (or self-destructive) personality disorder* | 17 | It is a habitual, stable, persistent, and pervasive pattern, with onset around age 8 but structurally evolving into adolescence and adulthood characterized by self-destructive tendencies, submissiveness, and high emotional sensibility:  17.1. self-destructive tendencies;  17.2. submissiveness and/or desires to be dominated (outside the  sexual realm);  17.3. high emotional sensitivity with the tendency to passive  manipulation;  17.4. unconscious pursuit of people and/or situations that may cause  disappointment and/or failure and/or living in distress and/or  mistreatment;  17.5. refusal to receive help and/or concrete support;  17.6. response to positive events with depression and guilt;  17.7. discomfort in the presence of pleasant and/or go-live situations;  17.8. inability to stay focused on assigned tasks;  17.9. withdrawal from any form of positive attention from others.  If the symptomatology is alternated with the sadistic pattern, with a greater or lesser prevalence, one must speak of "*sadomasochistic personality disorder*". | Latent domain (Cluster B) |
| *Psychopathic personality disorder* | 18 | It is a habitual, stable, persistent, and pervasive pattern, with onset around age 8 but evolves structurally into adolescence and adulthood characterized by antisocial behavior and narcissistic tendencies, manipulation, deficits in empathy, remorse, and guilt:  18.1. more or less overt antisocial behaviour;  18.2. deficit or absence of empathy;  18.3. absence of remorse, guilt and/or sense of shame;  18.4. egocentrism and/or marked propensity to impress the  interlocutor;  18.5. use of deception and/or manipulation to obtain personal benefits  and advantages;  18.6. impulsiveness and/or poor judgment;  18.7. irresponsibility and/or unreliability;  18.8. narcissistic tendencies;  18.9. poor or absent awareness of one's condition and/or emotions. | Psychotic domain (Cluster C) |
| *Schizophrenic personality disorder* | 19 | It is a habitual, stable, persistent, and pervasive pattern, with onset around age 10-12 but structurally evolving into adolescence and adulthood characterized by delusions, hallucinations, and disorganized speech:  19.1. delusions and hallucinations;  19.2. poor adherence to reality and little or no awareness of one's  schizophrenic state;  19.3. paranoia;  19.4. disorganized, incoherent and/or derailed speech;  19.5. coarse and disorganized and/or catatonic behaviour;  19.6. decrease in facial expressions and basic emotions, to the point  of abulia;  19.7. total or partial inability to take care of self and others;  19.8. extravagant and/or bizarre beliefs;  19.9. unusual or extremely irrational behavioural and/or perceptual  experiences.  "*Schizophreniform personality disorder*", currently codified in the DSM-V-TR, is characterized by symptoms identical to those of schizophrenia but with a duration of more than one month and less than 6 months, is considered here as an "*attenuated form of schizophrenic personality disorder*". | Psychotic domain (Cluster C) |
| *Schizoid personality disorder* | 20 | It is a habitual, stable, persistent, and pervasive pattern, with onset around age 10-12 but structurally evolving into adolescence and adulthood characterized by voluntary isolation, paranoia about human contact, and disinterest in social interaction:  20.1. difficulty and/or lack of desire to establish social  relationships;  20.2. voluntary isolation;  20.3. disinterest in sociality and/or strong interest in solitary activities;  20.4. poor adherence to reality and/or poor awareness of one's  psychotic state;  20.5. tendency toward paranoia;  20.6. nonexistent perceptions of threat;  20.7. affective flattening;  20.8. cold detachment from human relationships and/or absence of  close intimate relationships;  20.9. deep need to establish interpersonal spaces and limits of  sociability, even where there is no need. | Psychotic domain (Cluster C) |
| *Schizotypical personality disorder* | 21 | It is a habitual, stable, persistent, and pervasive pattern, with onset around age 10-12 but evolves structurally into adolescence and adulthood characterized by eccentric behaviors, bizarre beliefs, and unusual experiences without hallucinatory connotations:  21.1. eccentric behaviours;  21.2. bizarre and/or magical beliefs;  21.3. unusual perceptual experiences lacking hallucinatory  connotations;  21.4. prevalence of social detachment;  21.5. social discomfort;  21.6. rigid, reduced, restrained and/or context-inappropriate  affectivity;  21.7. use of deliberately unclear and/or metaphorical language;  21.8. little or absent awareness about one's emotions;  21.9. paranoid and/or obsessive thoughts. | Psychotic domain (Cluster C) |
| *Schizoaffective personality disorder* | 22 | It is a habitual, stable, persistent, and pervasive pattern, with onset around age 10-12 but evolving structurally into adolescence and adulthood characterized by schizophrenic symptoms, depressive symptoms, and extravagant and/or unusual beliefs:  22.1. delusions;  22.2. hallucinations;  22.3. poor adherence to reality and/or poor awareness of one's  psychotic state;  22.4. low tolerance for anxiety and/or frustration;  22.5. manic or hypomanic episodes;  22.6. inconstant and/or fluctuating mood;  22.7. bipolar tendency;  22.8. extravagant beliefs in the affective and relational sphere that  make relationships unstable;  22.9. unusual and/or extremely irrational behavioural and/or perceptual  experiences.  It is considered an intermediate form between depressive personality disorder and schizophrenic personality disorder. | Psychotic domain (Cluster C) |
| *Delusional personality disorder* | 23 | It is a habitual, stable, persistent, and pervasive pattern, with onset around age 8 but evolving structurally into adolescence and adulthood characterized by delusional beliefs, absence of persistent hallucinatory symptoms, and low or absent tolerance of criticism (relative to the delusional idea):  23.1. delusional belief enacted and/or prosecuted;  23.2. low or absent tolerance to criticism, judgment, anxiety and/or  frustration (relative to the delusional idea);  23.3. oddities and/or extravagances markedly detached from reality;  23.4. little adherence to reality and/or poor awareness of one's  delusional state;  23.5. absence of persistent hallucinations and/or irrelevant or  insignificant presence;  23.6. somatic symptoms, without clinical findings;  23.7. disorganized and/or coarse speech with poor adherence to reality;  23.8. conduct at the limits and/or beyond the normative dictate;  23.9. unrealistic ideas of a persecutory, relational, sentimental, somatic  and/or grandiose nature, then not pursued.  The condition does not have to meet the requirements for schizophrenic personality disorder, depressive personality disorder, and/or a specific medical condition (not attributable to psychopathologies). | Psychotic domain (Cluster C) |
| *Paranoid personality disorder* | 24 | It is a habitual, stable, persistent, and pervasive pattern, with onset around age 8 but evolving structurally into adolescence and adulthood characterized by a tendency toward paranoia, persecution mania, distrust, and suspiciousness:  24.1. tendency to paranoia;  24.2. persecution mania;  24.3. mistrust and suspiciousness;  24.4. unwarranted doubts;  24.5. low tolerance for anxiety and/or frustration;  24.6. prevalence of negative emotions and/or feelings;  24.7. phobias and/or obsessions;  24.8. tendency to social withdrawal;  24.9. refusal of confrontation and/or clarification if inconsistent with  his or her point of view, seeing the interlocutor as an enemy  and/or opponent. | Psychotic domain (Cluster C) |
| *Dissociative personality disorder* | 25 | It is a habitual, stable, persistent, and pervasive pattern with onset around age 8 but evolves structurally into adolescence and adulthood characterized by dissociative identity episodes, altered perception of reality, and somatic symptoms:  25.1. dissociative identity episodes;  25.2. altered perception of reality;  25.3. body-related somatic symptoms;  25.4. low tolerance to frustration and/or anxiety;  25.5. amnesic episodes;  25.6. psychotic episodes related to dissociation;  25.7. dissociative fugue (e.g., removal unexpectedly from the home  or wandering);  25.8. depersonalization episodes;  25.9. episodes of derealization. | Psychotic domain (Cluster C) |

**Supporting information 2**: Structural categories of the dysfunctional personality of Perrotta Integrative Clinica Interviews - 1 (children) -PICI-1C, with their respective characteristics

| ***Personality disorder*** | **N.** | **Definition and typologies** | **Dysfunctional area** |
| --- | --- | --- | --- |
| *Anxious disorder* | 1 | Please refer to its corresponding PICI-1TA, for definition and description, but it arises between 2 and 4 years of age | Neurotic domain (Cluster A) |
| *Phobic disorder* | 2 | Please refer to its corresponding PICI-1TA, for definition and description, but it arises between 2 and 4 years of age | Neurotic domain (Cluster A) |
| *Avoidant disorder* | 3 | Please refer to its corresponding PICI-1TA, for definition and description, but it arises between 2 and 4 years of age | Neurotic domain (Cluster A) |
| *Obsessive disorder* | 4 | Please refer to its corresponding PICI-1TA, for definition and description, but it arises between 2 and 4 years of age | Neurotic domain (Cluster A) |
| *Somatic disorder* | 5 | Please refer to its corresponding PICI-1TA, for definition and description, but it arises between 2 and 4 years of age | Neurotic domain (Cluster A) |
| *Manic disorder* | 6 | Please refer to its corresponding PICI-1TA, for definition and description, but it arises between 2 and 4 years of age | Neurotic domain (Cluster A) |
| *Bipolar disorder* | 7 | Please refer to its corresponding PICI-1TA, for definition and description, but it arises between 5 and 10 years of age | Latent domain (Cluster B) |
| *Disruptive mood dysregulation disorder* | 8 | It is a habitual, persistent and pervasive pattern, with onset between the ages of 5 and 10 years, characterized by systematic and persistent irritability involving angry outbursts, aggression and frequent mood swings:  8.1. severe outbursts of anger;  8.2. recurrent outbursts of anger, at least three episodes for  week;  8.3. violent physical and/or verbal reactions;  8.4. physical and/or verbal reactions disproportionate in both  duration and intensity;  8.5. angry and/or violent reactions inconsistent with age;  8.6. irritable mood for much of the day;  8.7. negative feelings directed toward the family, friendship  and/or school environment;  8.8. low tolerance for anxiety and/or frustration;  8.9. intolerance toward any form of education contrary to the  child's wishes and/or expectations; | Latent domain (Cluster B) |
| *Maladaptive separation disorder* | 9 | It is a habitual, persistent, and pervasive pattern, with onset between the ages of 2 and 4 years, characterized by systematic and persistent difficulty in letting go of parents or one's caregiver, constant and excessive fear that something tragic will happen to them, and systematic refusal to move away from home or stay alone in the home:  9.1. difficulty in letting go of parents and or their  caregiver;  9.2. outbursts of anger;  9.3. violent physical and/or verbal reactions;  9.4. physical and/or verbal reactions disproportionate in both  duration and intensity;  9.5. constant and/or excessive fear that something tragic will  happen to parents or their caregiver;  9.6. easily irritable, anxious and/or depressed mood (with  hints of apathy, restlessness and strong melancholy)  in the presence of a separating circumstance;  9.7. negative feelings directed toward the separative event;  9.8. low tolerance for anxiety and/or frustration;  9.9. systematic refusal to leave home and/or remain alone in  the home. | Latent domain (Cluster B) |
| *Oppositional-provocative disorder* | 10 | It is a habitual, persistent and pervasive pattern, with onset between the ages of 5 and 10 years, characterized by systemic and persistent difficulty in regulating and controlling one's emotions and behaviours:  10.1. choleric and/or easily irritable mood;  10.2. outbursts of anger;  10.3. violent physical and/or verbal reactions;  10.4. physical and/or verbal reactions disproportionate in both  duration and intensity;  10.5. oppositional behaviours;  10.6. vindictive behaviours;  10.7. negative feelings directed toward those in authority;  10.8. low tolerance for anxiety and/or frustration;  10.9. traits of hyperactivity. | Latent domain (Cluster B) |
| *Explosive-intermittent disorder* | 11 | It is a habitual, persistent, and pervasive pattern, with exor-dio between the ages of 4 and 8 years, characterized by systematic and persistent difficulty in managing anger and rage:  11.1) choleric and/or easily irritable mood;  11.2) outbursts of anger;  11.3) violent physical and/or verbal reactions;  11.4) physical and/or verbal reactions disproportionate in  both duration and intensity;  11.5) behaviours in reaction to events wrongly perceived as  damaging to one's sphere;  11.6) poor management of anger and/or rage, even in  completely harmless events;  11.7) negative feelings directed toward third parties;  11.8) low tolerance for anxiety and/or frustration;  11.9) poor ability to resist aggressive and/or violent impulses. | Latent domain (Cluster B) |
| *Uninhibited social engagement disorder* | 12 | It is a habitual, persistent and pervasive pattern, with onset between the ages of 5 and 10 years, characterized by the systematic and persistent display of behaviour, toward third parties, that is excessively physical and uninhibited:  12.1. unstable mood;  12.2. uninhibited verbal behaviours with people outside the  family unit;  12.3. direct and overly friendly approach with people not  belonging to the household;  12.4. uninhibited physical behaviour with people not  belonging to the household;  12.5. attention-seeking with outsiders or strangers;  12.6. the constant need for physical contact with people  outside the family unit;  12.7. overly trusting feelings directed toward third parties  (not previously known);  12.8. low tolerance of anxiety and/or frustration with respect  to seek contact and attention;  12.9. absence of reticence or hesitation to leave the safe place  with unknown persons. | Latent domain (Cluster B) |
| *Inhibited attachment disorder* | 13 | It is a habitual, persistent, and pervasive pattern, with exor-dio between the ages of two and five, which refers to the disturbed and/or inadequate social relational mode that characterizes the child relative to his or her level of psycho-social development, whether due to a distortion of the secure base:  13.1. difficulty in engaging in interpersonal relationships;  13.2. dysfunctional adaptation to common life circumstances;  13.3. excessive inhibition;  13.4. excessive hypervigilance;  13.5. contradictory attitude toward caregivers;  13.6. meagre social involvement;  13.7. difficulty in affective regulation;  13.8. low tolerance for anxiety and/or frustration;  13.9. unexplained fear and/or outbursts of anger. | Latent domain (Cluster B) |
| *Uninhibited attachment disorder* | 14 | It is a habitual, persistent, and pervasive pattern, with exor-dio between the ages of two and five, which refers to the disturbed and/or inadequate social relational mode that characterizes the child relative to his or her level of psycho-social development, whether due to a distortion of the secure base:  14.1. ease of engaging in interpersonal relationships;  14.2. independent and all-too-functional adaptation to  communal life circumstances;  14.3. excessive disinhibition;  14.4. excessive hypovigilance;  14.5. excessive detachment-seeking and separation from  caregivers;  14.6. excessive social involvement and/or excessive  sociability;  14.7. affective hyper regulation;  14.8. low tolerance of anxiety and/or frustration with respect  to loneliness;  14.9. absence of shyness toward the stranger with whom he  or she has contact. | Latent domain (Cluster B) |
| *Dependent disorder* | 15 | Please refer to its corresponding PICI-1TA, for definition and description, but it arises between 5 and 10 years of age | Latent domain (Cluster B) |
| *Depressive disorder* | 16 | Please refer to its corresponding PICI-1TA, for definition and description, but it arises between 5 and 10 years of age | Latent domain (Cluster B) |
| *Egoistic disorder* | 17 | It is a habitual, persistent and pervasive pattern, with a debut between the ages of 4 and 8 years, characterized by marked selfishness, emotional instability and sudden mood swings:  17.1. emotional instability;  17.2. sudden mood swings;  17.3. marked selfishness and/or empathy deficit;  17.4. desperate efforts to avoid estrangement and/or  abandonment (real and/or imagined);  17.5. inordinate need for attention to the detriment of others;  17.6. theatricality and/or drama;  17.7. active and/or passive-aggressive manipulation;  17.8. sudden anger and unwarranted aggression, with the use  of physical, verbal and/or psychological violence, with  or without aggression;  17.9. disregard for the rules and civil norms of coexistence | Latent domain (Cluster B) |
| *Libidic disorder* | 18 | It is a habitual, persistent and pervasive pattern, with a debut between the ages of 2 and 4 years, characterized by the inability to control libidinal drive and one's unconscious energies:  18.1. selfish tendencies and/or deficit of empathy;  18.2. manipulation of people and circumstances for personal  advantage, to the detriment of other people;  18.3. a marked sense of possession and ownership over people  and/or objects;  18.4. lacking or absent feeling of sharing;  18.5. high emotional sensitivity;  18.6. impatience and/or manic or hypomanic behaviour;  18.7. unconscious abuse of primitive defense mechanisms;  18.8. low tolerance for anxiety and/or frustration;  18.9. total or partial inability to resist impulses and desires,  to be realized immediately. | Latent domain (Cluster B) |
| *Psychopathic disorder* | 19 | It is a habitual, persistent and pervasive pattern, with a debut between the ages of 3 and 5 years, characterized by destructive and/or self-destructive tendencies, high emotional sensitivity and psychotic symptoms:  19.1. destructive and self-destructive tendencies;  19.2. high emotional sensitivity with a tendency for active  and/or passive manipulation and deception;  19.3. attention-seeking, to the detriment of others;  19.4. aggressive, violent actions and/or attitudes and/or in  violation of social and/or behavioural norms, more or  less manifested;  19.5. lack or absence of sensitivity, marked selfishness and/or  deficit of empathy;  19.6. absence or deficiency of remorse, guilt and/or sense of  shame;  19.7. self-centeredness;  19.8. excessive use of primitive defense mechanisms;  19.9. psychotic symptoms. | Psychotic domain (Cluster C) |

**Supporting information 3**: Neurotic Personality Disorder (NPD)

| *Specifications (of traits)* | *Types* | *Description* |
| --- | --- | --- |
| **Anxious** | *Monothematic* | The subject has been suffering for more than 6 months from a specific anxiety symptom (i.e., the primary cause that produced it is identified) that does not regress spontaneously and worsens quality of life |
|  | *Polythematic* | The subject is affected by more than 6 months of a series of specific anxious symptoms (i.e. we identify the primary causes that have produced them), involving one or more complex spheres of his existence (for example: if the social dimension is affected it will be a social anxiety, if the clinical dimension is affected it will be an anxiety of disease or mild hypochondria) that do not regress spontaneously and that worsen the quality of life without extending to all spheres of life of the subject |
|  | *Generalized* | The subject is affected for more than 6 months by the anxiety disorder of polythematic type but with extension to all spheres of life of the subject, producing a marked worsening quality of life. When the onset of symptoms is explosive, unmanageable will speak of generalized anxiety disorder from panic, while if it depends on a specific traumatic event will speak of post-traumatic stress disorder |
| **Phobic** | *Monothematic* | The subject has been suffering for more than 6 months from a specific phobic symptom (i.e., the primary cause that produced it is identified) that does not regress spontaneously and worsens the quality of life |
|  | *Polythematic* | The subject has been suffering for more than 6 months from a series of specific phobic symptoms (i.e. the primary causes that produced them are identified) that do not regress spontaneously, involving one or more complex spheres of their existence (i.e. if the social dimension is affected it will be a social phobia if the clinical dimension is affected it will be a phobia of disease or hypochondria) |
| **Avoidant** | *Monothematic* | The subject has been suffering for more than 6 months from a specific avoidant symptom (i.e., the primary cause that produced it is identified) that does not regress spontaneously and worsens quality of life |
|  | *Polythematic* | The subject has been suffering for more than 6 months from a series of specific avoidant symptoms (i.e. the primary causes that produced them are identified) that do not regress spontaneously, that involve one or more complex spheres of their existence (for example: if the social dimension is affected it will be a social avoidance, if the clinical dimension is affected it will be a hypochondriac avoidance) and that worsens the quality of life |
| **Somatic** | | The subject has been suffering for more than 6 months from a series of somatic specific/conversion symptoms (i.e., the primary causes that produced them are identified) or nonspecific/somatoform symptoms (i.e., the primary causes that produced them are not identified), which do not regress spontaneously, and which worsen the quality of life, in the absence of specific anxiety (because it would fall in the anxious personality disorder), phobia (because it would fall in the phobic disorder, as happens in hypochondria) or fictitious behaviours more or less voluntary (because it falls in the sphere of cluster B and/or C, as happens in Munchausen Syndrome) |
| **Obsessive** | *Monothematic* | The subject has been suffering for more than 6 months from a specific obsessive symptom (i.e. the primary cause that produced it is identified) that does not regress spontaneously and worsens the quality of life |
|  | *Polythematic* | The subject has been suffering for more than 6 months from a series of specific obsessive symptoms (i.e. the primary causes that produced them are identified) that do not regress spontaneously, and that involve one or more complex spheres of their existence |
|  | *Compulsive* | The subject is affected by more than 6 months of a series of specific obsessive symptoms (i.e. we identify the primary causes that have produced them) that do not regress spontaneously and that precede compensatory ritualistic behaviours (compulsions), able to negatively affect one or more complex spheres of their existence (eg body dysmorphism, compulsive accumulation, pyromania and compulsive self-injury type scratching other than the injurious forms present in the personality profiles of clusters B or C) |
| **Manic** | *Pure (type 1)* | The subject has been suffering for more than 6 months from a condition of classical manic hyperactivation determined by reduced need for sleep, increased talkativeness, flight of ideas and excessive self-esteem in the absence of symptoms typical of cluster B; symptoms that do not regress spontaneously, involving one or more complex spheres of their existence and worsening the quality of life |
|  | *Hypomanic (type 2)* | The subject has been suffering for more than 6 months from a condition of manic hypertension in an attenuated form (or hypomaniacality), which does not regress spontaneously, involving one or more complex spheres of his existence and worsening the quality of life, albeit in a lesser form than the classic form |

**Supporting information 4**: Computational corrections on the diagnosis of bipolarity, manic and depression

|  | **BIPOLAR** | **MANIC** | **DEPRESSIVE** | **FINAL DIAGNOSIS** |
| --- | --- | --- | --- | --- |
| **N_TRAITS** | 9  8  7  6 | <9  <8  <7  <6 | <9  <8  <7  <6 | *Bipolar* |
|  | 9  8  7  6 | 9  8  7  6 | <9  <8  <7  <6 | *Bipolar, markedly manic* |
|  | 9  8  7  6 | <9  <8  <7  <6 | 9  8  7  6 | *Bipolar, markedly depressive* |
|  | 5 | </=/>5 | </=/>5 | *Manic-depressive elevation*  *(mild bipolarity)* |
|  | <5 | 5-9 | < Manic | *Manic* |
|  | <5 | < Depressive | 5-9 | *Depressive* |
|  | 4 | 5-9 | 5-9 | *Manic-depressive predisposition*  *(humoral fragility)* |

**Supporting information 5**: Structured clinical interview for dysfunctional traits (Perrotta Integrative Clinical Interviews (PICI-TA), third version, of adolescents (14-18 years old), adults (19-69 years old) and elderly (70-90 years old)

| **SECTION A**  *Clinical interview for dysfunctional traits (PICI-TA-3), of adolescents (14-18 years), adults (19-69 years) and the elderly (70-90 years)* | | | |
| --- | --- | --- | --- |
| Operating note: Answer the questions, with the support of the trained therapist, choosing from 2 possible answers (Yes/No) and referring to your own experience of the last trimester of life. | | | |
| ***N*** | ***Items*** | ***Yes*** | ***No*** |
| *1* | Over the day, do you feel restless several times, with no real reason capable of justifying this emotional state? |  |  |
| *2* | Do you feel sometimes that you are too rigid in your positions? |  |  |
| *3* | Do you tend to complain for futile or apparent reasons? |  |  |
| *4* | Do you tend to stay focused on the same thought for too long? |  |  |
| *5* | Do your worries manifest themselves in repetitive thoughts? |  |  |
| *6* | Do you get irritated easily, without a serious enough reason to justify this emotional state? |  |  |
| *7* | Do you get mentally and/or physically tired easily? |  |  |
| *8* | Do you feel that daily worries or tensions are crushing you? |  |  |
| *9* | Do you feel that you can't finish all of your scheduled daily activities? |  |  |
| *10* | When you feel stressed do you indulge in hysterical behaviors? |  |  |
| *11* | When you feel stressed do you have obvious physical behaviors or symptoms? |  |  |
| *12* | Over the day, do you repeatedly feel tense and/or agitated, with no real reason capable of justifying these emotional states? |  |  |
| *13* | Do you have one or more unjustified fears? |  |  |
| *14* | Do you tend to avoid the source of your fear? |  |  |
| *15* | Do you tend to obsess or fixate on your fear? |  |  |
| *16* | When you are afraid do you tend not to confront the source that causes it? |  |  |
| *17* | Do you experience marked discomfort when you are in contact with the source of your fear? |  |  |
| *18* | Do you feel excessive and/or unfounded fear concerns a collective activity? |  |  |
| *19* | Do you tend to avoid the circumstance that causes you discomfort? |  |  |
| *20* | When you are afraid do you tend not to confront the source that causes it? |  |  |
| *21* | Do you perceive your self-esteem and confidence to be low? |  |  |
| *22* | Do you tend to avoid being involved in collective and/or public activities? |  |  |
| *23* | Do you feel discomfort and/or impatience when you have to perform collective and/or public activities? |  |  |
| *24* | Do you get affected by people's judgment of your performance? |  |  |
| *25* | Do you get affected by people's criticism of your performance? |  |  |
| *26* | Do you get conditioned by the rejections you receive from people about your performance? |  |  |
| *27* | Do you avoid taking risks, even if they are calculated? |  |  |
| *28* | Do you worry about receiving derision or ridicule for your mistakes? |  |  |
| *29* | Do you tend to obsessively fixate on an idea, object, or person? |  |  |
| *30* | Do you tend to have compulsive actions in reaction to your fixations/ obsessions? |  |  |
| *31* | Do you consider yourself a perfectionist or do you strive for perfection at all costs? |  |  |
| *32* | Do you feel better if you tend to control the circumstances of life or the actions of people with whom you relate? |  |  |
| *33* | Do you feel discomfort in public concerning your fixations and/or obsessions? |  |  |
| *34* | Do you worry about your state of health, even in the absence of obvious symptoms? |  |  |
| *35* | Have you ever felt that your body was different but that no one around you understood your state of mind concerning your perception? |  |  |
| *36* | Have you ever been convinced of something mistakenly but continued to believe it to be true? |  |  |
| *37* | Have you ever experienced one or more symptoms that were not explained by the doctors you consulted? |  |  |
| *38* | Have you ever consulted outside sources for your health problems, relying on people other than health professionals or qualified personnel, even after contacting them and gathering their professional opinions? |  |  |
| *39* | Do you feel that your mood is not always stable? |  |  |
| *40* | Do you feel that your ideas overlap? |  |  |
| *41* | Do you tend not to be frugal and/or spend excessively? |  |  |
| *42* | Do you feel that your ideas travel fast and/or leave one or more started tasks unfinished? |  |  |
| *43* | Do you feel excited several times a day? |  |  |
| *44* | Do you feel several times a day in need of human contact in public? |  |  |
| *45* | Do you feel several times a day need to lock yourself in your home or office to work, even well past working hours and/or cancelling appointments? |  |  |
| *46* | Do you think your ideas are brilliant or otherwise important? |  |  |
| *47* | Do you feel hyperactive at certain times of the day? |  |  |
| *48* | At certain times of the day do you have the feeling of being talkative? |  |  |
| *49* | Do you feel emotionally unstable? |  |  |
| *50* | Are your social relationships affected by your mood? |  |  |
| *51* | Have you ever felt, on the same day and at close times, both sad and elated? |  |  |
| *52* | Have you ever tried to actively obtain something against the other person's will? |  |  |
| *53* | Have you ever tried, with passive-aggressive attitudes, to get something against the other person's will? |  |  |
| *54* | Do you get hurt by criticism, even if it is deserved? |  |  |
| *55* | Do you tend to have more frequent unpleasant feelings and/or a tendentially pessimistic mode? |  |  |
| *56* | Do you ever enjoy voluntarily violating a social norm and/or civil commonality and not feeling sorry for yourself? |  |  |
| *57* | Have you ever suffered reprimands or punishments as a result of your behaviours? |  |  |
| *58* | Do you have difficulty getting in harmony with your emotions? |  |  |
| *59* | Have you ever had episodes of explosive and/or uncontrolled or otherwise unjustified anger concerning the event, then however compensated with guilt, shame or remorse? |  |  |
| *60* | Do you react to life events impulsively? |  |  |
| *61* | Do you react to life events with instinctiveness? |  |  |
| *62* | Do you experience physical and/or verbal anger and/or aggression toward people, objects, and/or animals? |  |  |
| *63* | Do you ever enjoy voluntarily violating a legal norm and not feeling sorry for it? |  |  |
| *64* | Do you feel more confident if you receive approval from others before starting an activity? |  |  |
| *65* | Do you have difficulty making daily decisions, even simple ones, and/or would you be able to make them on your own without asking for advice, suggestions, or help? |  |  |
| *66* | Do you have difficulty performing activities that would benefit and/or benefit you without asking for advice, suggestions and/or help? |  |  |
| *67* | Do you experience feelings of helplessness and/or discomfort when you are alone and/or cannot ask for advice, suggestions and/or help? |  |  |
| *68* | Do you feel excessive or unrealistic worry when you cannot ask for advice, suggestions and/or help? |  |  |
| *69* | Are you afraid to take care of yourself without someone's help? |  |  |
| *70* | Would you describe your mood as tending or always sad? |  |  |
| *71* | Do you perceive, over the day, one or more episodes of a marked decrease in pleasure in carrying out interests and activities? |  |  |
| *72* | Do you perceive, over the day, one or more episodes of marked boredom and/or disinterest, even though you have interesting activities that you can do? |  |  |
| *73* | Have you experienced weight loss and/or gain, as a result of your mood? |  |  |
| *74* | Have you experienced agitation and/or psychomotor slowdown as a result of your mood? |  |  |
| *75* | Do you frequently experience feelings of inappropriateness, self-evaluation, and/or marked guilt, in the absence of a justifiable cause? |  |  |
| *76* | Do you frequently experience negative or melancholic and/or death-related thoughts, unprovoked by actual events? |  |  |
| *77* | Do you feel that people, after getting to know you, tend to push you away and/or abandon you? |  |  |
| *78* | Do you do what you can, putting your all into it, to prevent people from pulling away from you and/or abandoning you? |  |  |
| *79* | Do you feel that your true being cannot come out externally and/or is better off not coming out and/or will not be understood if it comes out externally? |  |  |
| *80* | Do you perceive a sense of emptiness in yourself despite your daily activities and your family, friends, and work circle? |  |  |
| *81* | Have you ever been angry or aggressive unjustifiably and/or disproportionately to the offense or danger? |  |  |
| *82* | Have you ever had sudden anger without a specific trigger? |  |  |
| *83* | Have you ever been convinced of something irrational by believing it to be true and/or living it in your life as if it were a fact? |  |  |
| *84* | Have you ever heard voices and/or seen strange and/or bizarre things that others could not see and/or did not hear, believing them to be true even in the absence of further evidence? |  |  |
| *85* | Have you ever felt the need to attract attention to fill an inner void? |  |  |
| *86* | Do you feel uncomfortable when you are not the centre of attention? |  |  |
| *87* | Do you feel comfortable if you manifest your emotions with drama? |  |  |
| *88* | Do you feel comfortable if you manifest your emotions with theatricality and/or particular exaggeration? |  |  |
| *89* | Are you easily suggestible? |  |  |
| *90* | Do you sometimes use vague and/or impressionistic language to attract attention and/or narrate the events of your existence? |  |  |
| *91* | Do you consider your social contacts, in the personal and relational realm, to be unsafe and/or unstable and/or precarious and/or insecure? |  |  |
| *92* | Do you voluntarily use your body to attract attention? |  |  |
| *93* | Do you voluntarily use fascination and/or seduction and/or sexual techniques to attract attention? |  |  |
| *94* | Do you voluntarily use fascination and/or seductive and/or sexual techniques to manipulate situations and/or people to achieve your goals/aims? |  |  |
| *95* | Do you not care about and/or be affected by other people's life events, positive and/or negative, in that you cannot always understand other people's emotional positions? |  |  |
| *96* | Do you consider yourself a more special person than others and/or far above average? |  |  |
| *97* | Do you repute that your ideas are great and deserve a larger and more important stage? |  |  |
| *98* | Do you consider that you have very high self-esteem and that you come across as arrogant in other people's eyes? |  |  |
| *99* | Do you think others undeservedly envy your professional position and achievements? |  |  |
| *100* | Are you worried when you fantasize about your future and your ideas/thoughts of success? |  |  |
| *101* | Do you feel happy and satisfied when others look up to you and envy you? |  |  |
| *102* | Do you believe you deserve much more than you have, even if your qualifications and experience are not enough? |  |  |
| *103* | Do you use the idea of having low self-esteem to attract attention? |  |  |
| *104* | Do you feel that criticism and judgment manage to hurt you more than they should? |  |  |
| *105* | Do you tend to use complaints to get attention? |  |  |
| *106* | Do you tend to rattle others with your positions? |  |  |
| *107* | Despite your skills and qualities, do you feel that you do not deserve success? |  |  |
| *108* | Have you ever been told that you have narcissistic behaviours or attitudes? |  |  |
| *109* | Do you feel no remorse, guilt, or shame if you do wrong and/or have hurt someone or something? |  |  |
| *110* | Does it turn you on to see someone suffer? |  |  |
| *111* | Do you experience discomfort or negative feelings if you are in the presence of positive circumstances, situations, and/or feelings? |  |  |
| *112* | Does it turn you on to be the cause of someone's suffering? |  |  |
| *113* | Do you believe you have the right to make someone suffer without their consent? |  |  |
| *114* | Do you tend to identify the external with the internal and/or split the good and the bad? |  |  |
| *115* | Do you take pleasure in situations where you should feel suffering? |  |  |
| *116* | Do you feel suffering in situations where you should feel pleasure? |  |  |
| *117* | Do you tend to always ruin everything you are building up to something positive? |  |  |
| *118* | Do you like to submit and/or humiliate yourself outside the sexual sphere? |  |  |
| *119* | Do you seek out people and/or situations that may cause you disappointment and/or failure and/or live in discomfort and/or mistreatment? |  |  |
| *120* | If you are in trouble and know you need help, do you tend not to ask for help and/or avoid outside intervention by someone who could help you? |  |  |
| *121* | Do you feel sad and/or guilty if you experience good times? |  |  |
| *122* | Do you feel sad and/or guilty if you experience pleasant and goliardic moments? |  |  |
| *123* | Do you avoid whenever possible positive situations that might make you stand out in the eyes of others? |  |  |
| *124* | Do you tend to have deviant or criminal attitudes, even in the absence of legal consequences? |  |  |
| *125* | Do you tend to attract attention to appear? |  |  |
| *126* | Do you tend to attract attention by using impressionistic language? |  |  |
| *127* | Do you tend to be untrustworthy and/or irresponsible? |  |  |
| *128* | Are you aware that the reasons for your suffering depend on your behaviours, but yet you continue to repeat them? |  |  |
| *129* | Have you ever suffered from delusions and hallucinations? |  |  |
| *130* | Do you tend to convince yourself of a fact without ascertaining the genuineness of your interpretation? |  |  |
| *131* | Do you tend to believe that your interpretation is correct without ascertaining its genuineness? |  |  |
| *132* | Does your speech come across as disorganized, incoherent and/or derailed? |  |  |
| *133* | Does your behavior come across as coarse disorganized and/or catatonic? |  |  |
| *134* | Do your facial expressions and emotions tend toward Abulia? |  |  |
| *135* | Do you take little or no care of yourself? |  |  |
| *136* | Do you have ideas, beliefs, or thoughts that others find outlandish and/or bizarre? |  |  |
| *137* | Have you ever had unusual, strange and/or irrational experiences, behaviours and/or perceptions that others cannot explain? |  |  |
| *138* | Do you have difficulty and/or lack of desire in establishing social relationships? |  |  |
| *139* | Do you prefer voluntary isolation? |  |  |
| *140* | Do you experience disinterest in social interaction? |  |  |
| *141* | Do you feel a strong interest in solitary activities? |  |  |
| *142* | Do you tend to perceive facts, events, and/or people that are not threatening? |  |  |
| *143* | Do you feel that your emotions are increasingly cold and detached from social contexts? |  |  |
| *144* | Do you have few or no close emotional, sentimental and/or friendship relationships? |  |  |
| *145* | Do you have a deep need to establish interpersonal spaces and boundaries with other people, even where there is no need? |  |  |
| *146* | Do you have behaviors that others deem eccentric, and/or outlandish? |  |  |
| *147* | Do you have special and/or paranormal beliefs, powers, and/or psychic faculties? |  |  |
| *148* | Is social detachment the tendency you feel most comfortable with? |  |  |
| *149* | In the social setting do you feel uncomfortable and tense? |  |  |
| *150* | Do you perceive that when you are in a social setting your affectivity is diminished, more restrained, and/or inappropriate to the behaviour of others? |  |  |
| *151* | Do you prefer to make use of unclear and/or metaphor-rich language? |  |  |
| *152* | Do you feel that your thoughts tend to repeat themselves obsessively and/or become paranoid, in the absence of contrary and/or obvious evidence? |  |  |
| *153* | Have you ever had delusional behaviours enacted and/or pursued? |  |  |
| *154* | Have you ever suffered from hallucinations? |  |  |
| *155* | Have you ever suffered from manic or hypomanic episodes? |  |  |
| *156* | Have you ever suffered from bipolar tendencies? |  |  |
| *157* | Do you have a low or absent tolerance for criticism and/or judgment? |  |  |
| *158* | Does your speech come across as disorganized and/or coarse? |  |  |
| *159* | Have you ever had irrational ideas of persecutory, relational, sentimental, somatic and/or grandiose, without pursuing them? |  |  |
| *160* | Have you ever suffered from irrational beliefs or beliefs without a demonstrated or demonstrable basis, rigidity of thought and ideas full of anxiety and fear, which have produced distorted or otherwise wrong thoughts and judgements? |  |  |
| *161* | Have you ever suffered from paranoia (chronic delusion)? |  |  |
| *162* | Have you ever suffered from persecutory delusions? |  |  |
| *163* | Do you get assailed by doubts unjustifiably? |  |  |
| *164* | Have you ever suffered from phobias and/or obsessions? |  |  |
| *165* | Is social withdrawal the tendency you feel most comfortable with? |  |  |
| *166* | If someone has an opposing idea do you tend not to confront and/or see their opposition as a sign that they are an enemy? |  |  |
| *167* | Have you ever suffered from dissociative identity episodes? |  |  |
| *168* | Have you ever felt as if reality was not as you perceived it? |  |  |
| *169* | Have you ever suffered from amnestic episodes and/or memory lapses? |  |  |
| *170* | Have you ever seen hearing and/or seeing something or someone during your dissociation, that no one else heard and/or felt? |  |  |
| *171* | Have you ever wandered away from home or wandered around without (or partly) realizing that you were doing so? |  |  |
| *172* | Have you ever felt a feeling of disconnection from your body or your thoughts, to observe your life from the outside? |  |  |
| *173* | Have you ever felt a feeling of disconnection from your body or your thoughts, disassociating yourself from your surroundings? |  |  |

**Supporting information 6**: Structured clinical interview for dysfunctional traits (Perrotta Integrative Clinical Interviews - PICI-C), third version, of children (8-10 years) and preadolescents (11-13 years)

| **SECTION B**  *Clinical interview for dysfunctional traits (PICI-C-3),*  *of children (8-10 years) and preadolescents (11-13 years)* | | | |
| --- | --- | --- | --- |
| Operating Notes: Answer the questions, with the support of the trained therapist, choosing from 2 possible answers (Yes/No) and referring to your own experience of the last trimester of life. | | | |
| ***N*** | ***Items*** | ***Yes*** | ***No*** |
| *1* | Do you feel restless several times during the day, without any real reason capable of justifying this emotional state? |  |  |
| *2* | Do you feel, at times, that you are too rigid in your positions? |  |  |
| *3* | Do you tend to complain for futile or apparent reasons? |  |  |
| *4* | Do you tend to stay focused on the same thought for too long? |  |  |
| *5* | Do your worries manifest themselves in repetitive thoughts? |  |  |
| *6* | Do you get irritated easily, without a serious enough reason to justify this emotional state? |  |  |
| *7* | Do you tire mentally and/or physically with ease? |  |  |
| *8* | Do you feel daily worries or tensions crushing you? |  |  |
| *9* | Do you feel that you can't finish all of your scheduled daily activities? |  |  |
| *10* | When you feel stressed do you indulge in striking behaviors? |  |  |
| *11* | When you feel stressed do you have obvious physical behaviors or symptoms? |  |  |
| *12* | Over the day, do you feel restless, tense and agitated several times? |  |  |
| *13* | Do you have one or more unwarranted fears? |  |  |
| *14* | Do you tend to avoid the source of your fear? |  |  |
| *15* | Do you tend to obsess or fixate on your fear? |  |  |
| *16* | When you are afraid do you tend not to face the source that causes it? |  |  |
| *17* | Do you experience marked discomfort when you are in contact with the source of your fear? |  |  |
| *18* | Do you feel excessive and/or unfounded fear concerns a collective activity? |  |  |
| *19* | Do you tend to avoid the circumstance that causes you discomfort? |  |  |
| *20* | Do you tend to delegate your responsibility to someone else? |  |  |
| *21* | Do you perceive your self-esteem and confidence to be low? |  |  |
| *22* | Do you tend to avoid being involved in collective and/or public activities? |  |  |
| *23* | Do you feel discomfort and/or impatience when you have to perform collective and/or public activities? |  |  |
| *24* | Do you get affected by people's judgment of your performance? |  |  |
| *25* | Do you get affected by people's criticism of your performance? |  |  |
| *26* | Do you get conditioned by the rejections you receive from people about your performance? |  |  |
| *27* | Do you avoid taking risks, even if they are calculated? |  |  |
| *28* | Do you worry about receiving derision or ridicule for your mistakes? |  |  |
| *29* | Do you tend to obsessively fixate on an idea, object or person? |  |  |
| *30* | Do you tend to have compulsive actions in reaction to your fixations/ obsessions? |  |  |
| *31* | Do you consider yourself a perfectionist or do you strive for perfection at all costs? |  |  |
| *32* | Do you feel better if you tend to control the life circumstances or actions of the people with whom you relate? |  |  |
| *33* | Do you experience discomfort in public related to your fixations and/or obsessions? |  |  |
| *34* | Do you worry about your health status, even in the absence of obvious symptoms? |  |  |
| *35* | Have you ever felt that your body was different but that no one around you understood your state of mind about your perception? |  |  |
| *36* | Have you ever been convinced of something wrongly but continued to believe it to be true? |  |  |
| *37* | Have you ever experienced one or more symptoms that were not explained by the doctors you consulted? |  |  |
| *38* | Have you ever consulted outside sources for your health problems, relying on people other than health professionals or qualified personnel? |  |  |
| *39* | Do you feel that your mood is not always stable? |  |  |
| *40* | Do you feel that your ideas overlap? |  |  |
| *41* | Do you tend not to be frugal and/or overspend? |  |  |
| *42* | Do you feel that your ideas travel fast and/or leave one or more started activities unfinished? |  |  |
| *43* | Do you feel aroused several times a day? |  |  |
| *44* | Do you feel several times a day in need of human contact in public? |  |  |
| *45* | Do you feel several times a day in need of locking yourself in to think about your ideas? |  |  |
| *46* | Do you think your ideas are brilliant or otherwise important? |  |  |
| *47* | Do you feel hyperactive at certain times of the day? |  |  |
| *48* | At certain times of the day do you feel that you are logorrhoeic? |  |  |
| *49* | Do you feel emotionally unstable? |  |  |
| *50* | Are your social relationships affected by your mood? |  |  |
| *51* | Have you ever felt, on the same day and at close times, both depressed and elated? |  |  |
| *52* | Have you ever tried to actively obtain something against the other person's will? |  |  |
| *53* | Have you ever tried, with passive-aggressive attitudes, to obtain something against the other person's will? |  |  |
| *54* | Do you get hurt by criticism, even if it is deserved? |  |  |
| *55* | Do you tend to have more frequent unpleasant feelings and/or pessimistic ideas? |  |  |
| *56* | Have you ever had one or more egregious outbursts of anger? |  |  |
| *57* | Have you ever had recurrent striking outbursts of anger? |  |  |
| *58* | Have you ever had violent physical and/or verbal reactions? |  |  |
| *59* | Have you ever had disproportionate physical and/or verbal reactions? |  |  |
| *60* | Have you ever had egregious violent reactions? |  |  |
| *61* | Have you ever had negative feelings directed toward the family, friendship and/or school environment? |  |  |
| *62* | Have you ever had episodes of intolerance toward one or more forms of education because they were perceived by you to be contrary to your wishes and/or expectations? |  |  |
| *63* | Have you ever had difficulty letting go of those who cared for you? |  |  |
| *64* | Have you ever been afraid that something tragic would happen to your caregiver and that it would make you deeply ill? |  |  |
| *65* | Have you ever felt irritated, depressed, or anxious in anticipation of a temporary separation from caregivers? |  |  |
| *66* | Have you ever experienced negative feelings coinciding with an event of temporary separation from those you love and/or caregivers? |  |  |
| *67* | Have you ever refused to stay home alone? |  |  |
| *68* | Have you ever forced your caregivers to stay there and not leave, even if unnecessary? |  |  |
| *69* | Have you ever forcefully and violently resisted an order from a caregiver? |  |  |
| *70* | Have you ever retaliated against an order from a caregiver? |  |  |
| *71* | Have you ever experienced negative feelings coinciding with a request, order, or command from a caregiver or authority? |  |  |
| *72* | Have you ever felt the need to react with anger, even in the face of completely harmless events? |  |  |
| *73* | Have you ever decided to react with anger, even though you knew the events were entirely harmless? |  |  |
| *74* | Have you ever decided on the overwhelming urge to react in anger, even though you knew the events were all harmless or merely annoying? |  |  |
| *75* | Have you ever engaged in uninhibited verbal behaviour with people outside the family unit? |  |  |
| *76* | Have you ever engaged in uninhibited physical behaviour with people outside the household? |  |  |
| *77* | Have you ever had a direct and overly friendly approach with people outside the family unit? |  |  |
| *78* | Have you ever sought attention from strangers or unknown people? |  |  |
| *79* | Have you ever had a constant need for physical contact with people outside the family unit? |  |  |
| *80* | Have you ever had overly trusting feelings directed toward third parties (not previously known)? |  |  |
| *81* | Have you ever been tense and nervous if you could not seek contact or attention from people outside your family? |  |  |
| *82* | Have you ever taken pleasure in straying with strangers or people outside your household without notifying your parents or caregivers? |  |  |
| *83* | Have you ever had difficulty communicating with other children? |  |  |
| *84* | Have you ever had difficulty communicating with adults? |  |  |
| *85* | Have you ever felt that you were held back in your relationships with other people? |  |  |
| *86* | Have you ever been overly concerned about a conversation with someone? |  |  |
| *87* | Do you ever have unkind attitudes toward caregivers? |  |  |
| *88* | Do you tend to avoid contact with other people in public or situations where you are in public? |  |  |
| *89* | Have you ever had difficulty in knowing how to manage your emotions? |  |  |
| *90* | Have you ever had negative feelings or fears toward someone or something without knowing them? |  |  |
| *91* | Have you ever had an ease in engaging in interpersonal relationships? |  |  |
| *92* | Have you ever had an easy time adapting to life's circumstances? |  |  |
| *93* | Have you ever had overly exuberant and uninhibited behaviour with adults? |  |  |
| *94* | Have you ever had overly suspicious and/or protective attitudes toward yourself when you had to relate to other people outside your family circle? |  |  |
| *95* | Have you ever had attitudes of excessive detachment and separation from caregivers? |  |  |
| *96* | Have you ever had excessive social involvement and/or excessive sociability? |  |  |
| *97* | Have you ever had an overly exaggerated emotional display? |  |  |
| *98* | Have you ever felt irritated, depressed, or anxious at the idea of being alone, even though your desire was sociability? |  |  |
| *99* | Have you ever had an absence of shyness in the presence of a stranger on first contact? |  |  |
| *100* | Do you feel more confident if you receive approval from others before starting an activity? |  |  |
| *101* | Do you have difficulty making daily decisions, even simple ones, and/or would you be able to make them on your own, without asking for advice, suggestion, or help? |  |  |
| *102* | Do you have difficulty performing activities that would benefit and/or benefit you, without asking for advice, suggestions and/or help? |  |  |
| *103* | Do you experience feelings of helplessness and/or discomfort when you are alone and/or cannot ask for advice, suggestions, and/or help? |  |  |
| *104* | Do you experience excessive or unrealistic worry when you cannot ask for advice, suggestions and/or help? |  |  |
| *105* | Are you afraid to take care of yourself without someone's help? |  |  |
| *106* | Would you describe your mood as tending or always depressed? |  |  |
| *107* | Do you perceive, over the day, one or more episodes of markedly decreased pleasure in pursuing interests and activities? |  |  |
| *108* | Do you perceive, over the day, one or more episodes of marked boredom and/or disinterest, even though you have interesting activities you can do? |  |  |
| *109* | Have you experienced weight loss and/or gain as a result of your mood? |  |  |
| *110* | Have you experienced agitation and/or psychomotor slowdown as a result of your mood? |  |  |
| *111* | Do you frequently experience feelings of inappropriateness, self-evaluation, and/or marked guilt, in the absence of a justifiable cause? |  |  |
| *112* | Do you frequently experience negative or melancholic and/or death-related thoughts, unprovoked by actual events? |  |  |
| *113* | Do you wish other people would always do what you wish? |  |  |
| *114* | Do you think that people, after getting to know you, mostly want to abandon you or push you away? |  |  |
| *115* | Would you like it if other people gave you attention even if to someone else's detriment? |  |  |
| *116* | When frustrated and/or under tension do you like to attract attention with drama and theatrics? |  |  |
| *117* | Have you ever reacted with sudden anger and unwarranted aggression, with/without the use of physical, verbal and/or psychological violence? |  |  |
| *118* | Have you ever willfully violated one or more civil rules of coexistence and/or legal rules? |  |  |
| *119* | Do you refuse to lend, even temporarily, items you care about to other people out of jealousy and/or possession? |  |  |
| *120* | Do you refuse to share items you care about, with other people, out of jealousy and/or possession? |  |  |
| *121* | Do you consider yourself an impatient, restless and/or overly hurried person? |  |  |
| *122* | Do you tend to identify the external with the internal and/or split the good and the bad? |  |  |
| *123* | Do you feel as irrepressible and/or irrepressible as the desire to achieve what you think and/or desire? |  |  |
| *124* | If something can't be yours, would you rather destroy it so someone else doesn't have it? |  |  |
| *125* | Have you ever had aggressive, violent actions and/or attitudes and/or in violation of social norms and/or behaviour, more or less manifested? |  |  |
| *126* | Do you feel disinterest and/or irritation about what the other person is feeling emotionally and/or sentimentally, even if he or she is suffering and/or struggling? |  |  |
| *127* | Do you feel remorse, guilt, or a sense of shame if you do something wrong? |  |  |
| *128* | Have you ever sensed strange creatures, mysterious beings, voices and/or sounds that no one else could see or hear? |  |  |

**Supporting information 7**: Structured clinical interview (Perrotta Integrative Clinical Interviews - PICI-SD) for common secondary disorders of all developmental ages investigated by PICI (8-90 years)

| **SECTION C**  *Clinical interview for secondary disorders (PICI-SD-3) of all developmental ages (8-90 years)* | | | |
| --- | --- | --- | --- |
| Operating Notes: Answer the questions, with the support of the trained therapist, choosing from 2 possible answers (Yes/No) and referring to your own experience of the last half year of life. | | | |
| ***N*** | ***Items*** | ***Yes*** | ***No*** |
| *1* | Have you been diagnosed with or have obvious symptoms of a *neurodevelopmental disorder* for at least 6 months? If yes, please specify the type:  a. Intellectual disability  b. Global developmental delay  c. Generalized developmental disorder, not otherwise specified  d. Specific language disorder: phonological decoding  e. Specific language disorder: encoding  f. Specific language disorder: morphological decoding  g. Specific language disorder: syntactic decoding  h. Specific language disorder: processing  i. Communication disorder: phonetic-phonological (or articulation)  j. Communication disorder: verbal fluency (cluttering type)  k. Communication disorder: verbal fluency (stuttering type)  l. Communication disorder: social (or pragmatic)  m. Autistic spectrum  n. Attention-deficit/hyperactivity disorder (ADHD) - type inattention  o. Attention-deficit/hyperactivity disorder (ADHD) - hyperactivity type  p. Specific learning disorders (SLDs) - dyslexia  q. Specific learning disorders (SLDs) - Dysgraphia/Dysorthographia  r. Specific learning disorders (SLDs) - Dyscalculia  s. Specific learning disorders (SLDs) - Computation  t. Movement disorders: coordination  u. Movement disorders: stereotyped movement  v. Movement disorders: persistent (chronic) / transient (acute) TIC  x. Movement disorders: Tourette's |  |  |
| *2* | Have you been diagnosed with or are there obvious symptoms of an acute or *short-lived psychotic disorder*, even a single one? If yes, specify in the last three years how many episodes and describe their symptoms and duration  ____________________________________________________________  ____________________________________________________________ |  |  |
| *3* | Have you been diagnosed with or are there obvious symptoms of a *catatonic event*, even a single one? If yes, specify in the last three years how many episodes and describe their symptoms and duration  ____________________________________________________________  ____________________________________________________________ |  |  |
| *4* | Have you been diagnosed with or have obvious symptoms of selective *mutism disorder* for at least 1 month, in the age group 2-18 years? If yes, specify how many episodes and describe their symptoms and duration  ____________________________________________________________  ____________________________________________________________ |  |  |
| *5* | Have you been diagnosed with or have obvious symptoms of a *nutrition disorder* for at least 2 months? If yes, please specify the type:  a. Picacism disorder  b. Rumination disorder  c. Avoidant-restrictive food intake disorder  d. Anorexia nervosa  e. Bulimia nervosa  f. Uncontrolled eating disorder (binge eating) |  |  |
| *6* | Have you been diagnosed with or have obvious symptoms of an *evacuation disorder* been present for at least 1 month (for subjects younger than age 10 years) or have they been present in childhood (for subjects older than age 10 years)? If yes, specify the type:  a. Enuresis  b. Encopresis |  |  |
| *7* | Have you been diagnosed with or have obvious symptoms of a *sleep-wake disorder* for at least 3 months? If yes, please specify the type:  a. Insomnia disorder  b. Hypersomnolence/Narcolepsy disorder  c. Breathing-related disorders: apnea/ obstructive sleep hypopnea/ snoring /  awakenings (own or others) as a result of snoring / sleep-related  hypoventilation  d. Parasomnia: confusional awakenings  e. Parasomnia: sleepwalking  f. Parasomnia: night terrors  g. Parasomnia: exploding head syndrome  h. Parasomnia: nocturnal agitation  i. Parasomnia: nocturnal paralysis  j. Parasonnia: night terrors  k. Parasonnia: hypnagogic/hypnopompic hallucinations  l. Parasomnia: sleep-related eating disorder  m. Parasonnia: sexsomnia  n. Other parasomnias/other sleep-related disorders |  |  |
| *8* | Have you been diagnosed with or have obvious symptoms of a *gender identity disorder (or gender dysphoria)* for at least 6 months? If yes, please specify the type:  a. Gender dysphoria without transition  b. Gender dysphoria in transition  c. Gender dysphoria with transition completed |  |  |
| *9* | Have you been diagnosed with or have obvious symptoms of a *sexual dimension disorder* for at least 3 months? If yes, please specify the type:  a. Paraphiliac disorder  b. Psychological sexual dysfunction  c. Hormonal sexual dysfunction  d. Mechanical/functional sexual dysfunctions  e. Sexual dysfunction as a result of traumatic event  f. Sexual dysfunction as a result of tumour event  g. Mixed sexual dysfunction or other causes not specified in the list |  |  |
| *10* | Have you been diagnosed with or have obvious symptoms of an *alcohol and/or drug dependence disorder* for at least 3 months or at least weekly use? If yes, please specify:  a. Alcohol  b. Narcotic substance  c. Tobacco  d. Other substance (specify):_____________________________________ |  |  |
| *11* | Have you been diagnosed with or have obvious symptoms of a *behavioural addiction disorder* for at least 3 months? If yes, please specify:  a. Gambling  b. Emotional and/or compulsive shopping  c. Technology, internet and social networking  d. Gaming  e. Work  f. Sports  g. Sex  h. Emotional relationships  i. Unjustified dietary restrictions (orthorexia)  j. Other behavioral addictions not listed (specify): _____________________ |  |  |
| *12* | Have you been diagnosed with or have obvious symptoms of *suicidal tendency* been present for at least 1 week, during the lifetime course? If yes, please specify:  a. Suicidal thinking, not manifested but idealized and without intention  concrete intention to carry it out  b. Suicidal thinking, not manifested but idealized and with intention concrete  intention to carry it out even though it has not yet been implemented  c. Suicidal thinking, idealized and manifested to other people with the  purpose of complaining  d. Suicidal thinking, idealized and manifested to other people with the  purpose of attracting attention and causing concern and alarm  e. Self-injurious physical manifestation without suicidal purpose  f. Self-injurious physical manifestation with incomplete suicidal purpose  g. Self-injurious physical manifestation with complete suicidal purpose but  not realized  h. Self-injurious physical manifestation with suicidal purpose complete and  realized, resulting in minor injury to self and/or others  i. Self-injurious physical manifestation with suicidal purpose complete and  realized, which resulted in serious injury to self and/or others  j. Self-injurious physical manifestation with suicidal purpose complete and  realized, which resulted in very serious injury to self and/or others |  |  |
| Other relevant clinical notes:  ___________________________________________________________________________  ___________________________________________________________________________  ___________________________________________________________________________  ___________________________________________________________________________  ___________________________________________________________________________  ___________________________________________________________________________  ___________________________________________________________________________ | | | |

**Supporting information 8**: Structured clinical interview for functional traits (Perrotta Integrative Clinical Interviews - PICI-FT), third version, for all developmental stages investigated by PICI (8-90 years)

| **SECTION D**  *Clinical interview for functional traits (PICI-FT-3),*  *for all developmental ages (8-90 years)* | | |
| --- | --- | --- |
| Operating Notes: Answer the questions, with the support of the trained therapist, choosing from 6 possible answers (L0-5 scale, in which "0" corresponds to "never" and "5" corresponds to "always") and referring to one's personal experience of the last year of life. | | |
| **N** | **AREAS OF OPERATION** | **BEHAVIORAL STYLES** |
| 1 | Courage | \| 1.1. Timorous  1.2. Doubting  1.3. Sure  1.4. Adventurous  1.5. Fearless \| \| **0** \| **1** \| **2** \| **3** \| **4** \| **5** \| **______** \| \| --- \| --- \| --- \| --- \| --- \| --- \| --- \| \| **0** \| **1** \| **2** \| **3** \| **4** \| **5** \| \| **0** \| **1** \| **2** \| **3** \| **4** \| **5** \| \| **0** \| **1** \| **2** \| **3** \| **4** \| **5** \| \| **0** \| **1** \| **2** \| **3** \| **4** \| **5** \| \| \| --- \| --- \| --- \| --- \| --- \| --- \| --- \| --- \| --- \| --- \| --- \| --- \| --- \| --- \| --- \| --- \| --- \| --- \| --- \| --- \| --- \| --- \| --- \| --- \| --- \| --- \| --- \| --- \| --- \| --- \| --- \| --- \| --- \| \|  \|  \| |
| 2 | Intrapreneurship | \| 2.1. Cheeky  2.2. Spontaneous  2.3. Clear  2.4. Sincere  2.5. Direct \| \| \| **0** \| **1** \| **2** \| **3** \| **4** \| **5** \| **______** \| \| --- \| --- \| --- \| --- \| --- \| --- \| --- \| \| **0** \| **1** \| **2** \| **3** \| **4** \| **5** \| \| **0** \| **1** \| **2** \| **3** \| **4** \| **5** \| \| **0** \| **1** \| **2** \| **3** \| **4** \| **5** \| \| **0** \| **1** \| **2** \| **3** \| **4** \| **5** \| \| \| --- \| --- \| --- \| --- \| --- \| --- \| --- \| --- \| --- \| --- \| --- \| --- \| --- \| --- \| --- \| --- \| --- \| --- \| --- \| --- \| --- \| --- \| --- \| --- \| --- \| --- \| --- \| --- \| --- \| --- \| --- \| --- \| --- \| --- \| \|  \|  \| \| |
| 3 | Self-control | \| 3.1. Hypercontroller  3.2. Moderate  3.3. Diligent  3.4. Free  3.5. Hypocontroller \| \| **0** \| **1** \| **2** \| **3** \| **4** \| **5** \| **______** \| \| --- \| --- \| --- \| --- \| --- \| --- \| --- \| \| **0** \| **1** \| **2** \| **3** \| **4** \| **5** \| \| **0** \| **1** \| **2** \| **3** \| **4** \| **5** \| \| **0** \| **1** \| **2** \| **3** \| **4** \| **5** \| \| **0** \| **1** \| **2** \| **3** \| **4** \| **5** \| \| \| --- \| --- \| --- \| --- \| --- \| --- \| --- \| --- \| --- \| --- \| --- \| --- \| --- \| --- \| --- \| --- \| --- \| --- \| --- \| --- \| --- \| --- \| --- \| --- \| --- \| --- \| --- \| --- \| --- \| --- \| --- \| --- \| --- \| \|  \|  \| |
| 4 | Energy | \| 4.1. Liabilities  4.2. Tiepid  4.3. Harmonious  4.4. Energetic  4.5. Active \| \| **0** \| **1** \| **2** \| **3** \| **4** \| **5** \| **______** \| \| --- \| --- \| --- \| --- \| --- \| --- \| --- \| \| **0** \| **1** \| **2** \| **3** \| **4** \| **5** \| \| **0** \| **1** \| **2** \| **3** \| **4** \| **5** \| \| **0** \| **1** \| **2** \| **3** \| **4** \| **5** \| \| **0** \| **1** \| **2** \| **3** \| **4** \| **5** \| \| \| \| --- \| --- \| --- \| --- \| --- \| --- \| --- \| --- \| --- \| --- \| --- \| --- \| --- \| --- \| --- \| --- \| --- \| --- \| --- \| --- \| --- \| --- \| --- \| --- \| --- \| --- \| --- \| --- \| --- \| --- \| --- \| --- \| --- \| --- \| \|  \| \|  \| |
| 5 | Security | \| 5.1. Insecure  5.2. Uncertain  5.3. Responsible  5.4. Reliable  5.5. Safe \| \| **0** \| **1** \| **2** \| **3** \| **4** \| **5** \| **______** \| \| --- \| --- \| --- \| --- \| --- \| --- \| --- \| \| **0** \| **1** \| **2** \| **3** \| **4** \| **5** \| \| **0** \| **1** \| **2** \| **3** \| **4** \| **5** \| \| **0** \| **1** \| **2** \| **3** \| **4** \| **5** \| \| **0** \| **1** \| **2** \| **3** \| **4** \| **5** \| \| \| \| --- \| --- \| --- \| --- \| --- \| --- \| --- \| --- \| --- \| --- \| --- \| --- \| --- \| --- \| --- \| --- \| --- \| --- \| --- \| --- \| --- \| --- \| --- \| --- \| --- \| --- \| --- \| --- \| --- \| --- \| --- \| --- \| --- \| --- \| \|  \| \|  \| |
| 6 | Dexterity | \| 6.1. Unprovided  6.2. Naive  6.3. Careful  6.4. Astute  6.5. Shrewd \| \| **0** \| **1** \| **2** \| **3** \| **4** \| **5** \| **______** \| \| --- \| --- \| --- \| --- \| --- \| --- \| --- \| \| **0** \| **1** \| **2** \| **3** \| **4** \| **5** \| \| **0** \| **1** \| **2** \| **3** \| **4** \| **5** \| \| **0** \| **1** \| **2** \| **3** \| **4** \| **5** \| \| **0** \| **1** \| **2** \| **3** \| **4** \| **5** \| \| \| \| --- \| --- \| --- \| --- \| --- \| --- \| --- \| --- \| --- \| --- \| --- \| --- \| --- \| --- \| --- \| --- \| --- \| --- \| --- \| --- \| --- \| --- \| --- \| --- \| --- \| --- \| --- \| --- \| --- \| --- \| --- \| --- \| --- \| --- \| \|  \| \|  \| |
| 7 | Sociability | \| 7.1. Rigid  7.2. Accommodating  7.3. Sociable  7.4. Friendly  7.5. Welcoming \| \| **0** \| **1** \| **2** \| **3** \| **4** \| **5** \| **______** \| \| --- \| --- \| --- \| --- \| --- \| --- \| --- \| \| **0** \| **1** \| **2** \| **3** \| **4** \| **5** \| \| **0** \| **1** \| **2** \| **3** \| **4** \| **5** \| \| **0** \| **1** \| **2** \| **3** \| **4** \| **5** \| \| **0** \| **1** \| **2** \| **3** \| **4** \| **5** \| \| \| \| --- \| --- \| --- \| --- \| --- \| --- \| --- \| --- \| --- \| --- \| --- \| --- \| --- \| --- \| --- \| --- \| --- \| --- \| --- \| --- \| --- \| --- \| --- \| --- \| --- \| --- \| --- \| --- \| --- \| --- \| --- \| --- \| --- \| --- \| \|  \| \|  \| |
| 8 | Passionately | \| 8.1. Indifferent  8.2. Detached  8.3. Involved  8.4. Passionate  8.5. Fiery \| \| **0** \| **1** \| **2** \| **3** \| **4** \| **5** \| **______** \| \| --- \| --- \| --- \| --- \| --- \| --- \| --- \| \| **0** \| **1** \| **2** \| **3** \| **4** \| **5** \| \| **0** \| **1** \| **2** \| **3** \| **4** \| **5** \| \| **0** \| **1** \| **2** \| **3** \| **4** \| **5** \| \| **0** \| **1** \| **2** \| **3** \| **4** \| **5** \| \| \| \| --- \| --- \| --- \| --- \| --- \| --- \| --- \| --- \| --- \| --- \| --- \| --- \| --- \| --- \| --- \| --- \| --- \| --- \| --- \| --- \| --- \| --- \| --- \| --- \| --- \| --- \| --- \| --- \| --- \| --- \| --- \| --- \| --- \| --- \| \|  \| \|  \| |
| 9 | Sensitivity | \| 9.1. Hyposensitive  9.2. Disinterested  9.3. Sensitive  9.4. Interested  9.5. Hypersensitive \| \| **0** \| **1** \| **2** \| **3** \| **4** \| **5** \| **______** \| \| --- \| --- \| --- \| --- \| --- \| --- \| --- \| \| **0** \| **1** \| **2** \| **3** \| **4** \| **5** \| \| **0** \| **1** \| **2** \| **3** \| **4** \| **5** \| \| **0** \| **1** \| **2** \| **3** \| **4** \| **5** \| \| **0** \| **1** \| **2** \| **3** \| **4** \| **5** \| \| \| --- \| --- \| --- \| --- \| --- \| --- \| --- \| --- \| --- \| --- \| --- \| --- \| --- \| --- \| --- \| --- \| --- \| --- \| --- \| --- \| --- \| --- \| --- \| --- \| --- \| --- \| --- \| --- \| --- \| --- \| --- \| --- \| --- \| \|  \|  \| |
| 10 | Emotionality | \| 10.1. Transparent  10.2. Limpid  10.3. Adapted  10.4. Protected  10.5. Opaque \| \| **0** \| **1** \| **2** \| **3** \| **4** \| **5** \| **______** \| \| --- \| --- \| --- \| --- \| --- \| --- \| --- \| \| **0** \| **1** \| **2** \| **3** \| **4** \| **5** \| \| **0** \| **1** \| **2** \| **3** \| **4** \| **5** \| \| **0** \| **1** \| **2** \| **3** \| **4** \| **5** \| \| **0** \| **1** \| **2** \| **3** \| **4** \| **5** \| \| \| \| --- \| --- \| --- \| --- \| --- \| --- \| --- \| --- \| --- \| --- \| --- \| --- \| --- \| --- \| --- \| --- \| --- \| --- \| --- \| --- \| --- \| --- \| --- \| --- \| --- \| --- \| --- \| --- \| --- \| --- \| --- \| --- \| --- \| --- \| \|  \| \|  \| |
| 11 | Relationality  (relationships with others) | \| 11.1. Superficial  11.2. Fickle  11.3. Reliable  11.4. Methodical  11.5. Rigid \| \| **0** \| **1** \| **2** \| **3** \| **4** \| **5** \| **______** \| \| --- \| --- \| --- \| --- \| --- \| --- \| --- \| \| **0** \| **1** \| **2** \| **3** \| **4** \| **5** \| \| **0** \| **1** \| **2** \| **3** \| **4** \| **5** \| \| **0** \| **1** \| **2** \| **3** \| **4** \| **5** \| \| **0** \| **1** \| **2** \| **3** \| **4** \| **5** \| \| \| \| --- \| --- \| --- \| --- \| --- \| --- \| --- \| --- \| --- \| --- \| --- \| --- \| --- \| --- \| --- \| --- \| --- \| --- \| --- \| --- \| --- \| --- \| --- \| --- \| --- \| --- \| --- \| --- \| --- \| --- \| --- \| --- \| --- \| --- \| \|  \| \|  \| |
| 12 | Affectivity | \| 12.1. Employee  12.2. Subordinate  12.3. Self-employed  12.4. Independent  12.5. Individualist \| \| **0** \| **1** \| **2** \| **3** \| **4** \| **5** \| **______** \| \| --- \| --- \| --- \| --- \| --- \| --- \| --- \| \| **0** \| **1** \| **2** \| **3** \| **4** \| **5** \| \| **0** \| **1** \| **2** \| **3** \| **4** \| **5** \| \| **0** \| **1** \| **2** \| **3** \| **4** \| **5** \| \| **0** \| **1** \| **2** \| **3** \| **4** \| **5** \| \| \| \| --- \| --- \| --- \| --- \| --- \| --- \| --- \| --- \| --- \| --- \| --- \| --- \| --- \| --- \| --- \| --- \| --- \| --- \| --- \| --- \| --- \| --- \| --- \| --- \| --- \| --- \| --- \| --- \| --- \| --- \| --- \| --- \| --- \| --- \| \|  \| \|  \| |
| 13 | Altruism | \| 13.1. Selfish  13.2. Defensive  13.3. Available  13.4. Good  13.5. Generous \| \| **0** \| **1** \| **2** \| **3** \| **4** \| **5** \| **______** \| \| --- \| --- \| --- \| --- \| --- \| --- \| --- \| \| **0** \| **1** \| **2** \| **3** \| **4** \| **5** \| \| **0** \| **1** \| **2** \| **3** \| **4** \| **5** \| \| **0** \| **1** \| **2** \| **3** \| **4** \| **5** \| \| **0** \| **1** \| **2** \| **3** \| **4** \| **5** \| \| \| \| --- \| --- \| --- \| --- \| --- \| --- \| --- \| --- \| --- \| --- \| --- \| --- \| --- \| --- \| --- \| --- \| --- \| --- \| --- \| --- \| --- \| --- \| --- \| --- \| --- \| --- \| --- \| --- \| --- \| --- \| --- \| --- \| --- \| --- \| \|  \| \|  \| |
| 14 | Fantasy | \| 14.1. abstract  14.2. Artistic  14.3. Symmetrical  14.4. methodical  14.5. Concrete \| \| **0** \| **1** \| **2** \| **3** \| **4** \| **5** \| **______** \| \| --- \| --- \| --- \| --- \| --- \| --- \| --- \| \| **0** \| **1** \| **2** \| **3** \| **4** \| **5** \| \| **0** \| **1** \| **2** \| **3** \| **4** \| **5** \| \| **0** \| **1** \| **2** \| **3** \| **4** \| **5** \| \| **0** \| **1** \| **2** \| **3** \| **4** \| **5** \| \| \| \| --- \| --- \| --- \| --- \| --- \| --- \| --- \| --- \| --- \| --- \| --- \| --- \| --- \| --- \| --- \| --- \| --- \| --- \| --- \| --- \| --- \| --- \| --- \| --- \| --- \| --- \| --- \| --- \| --- \| --- \| --- \| --- \| --- \| --- \| \|  \| \|  \| |
| 15 | Morality | \| 15.1. Transgressive  15.2. Alternative  15.3. Cooperative  15.4. Collaborative  15.5. Compliant \| \| **0** \| **1** \| **2** \| **3** \| **4** \| **5** \| **______** \| \| --- \| --- \| --- \| --- \| --- \| --- \| --- \| \| **0** \| **1** \| **2** \| **3** \| **4** \| **5** \| \| **0** \| **1** \| **2** \| **3** \| **4** \| **5** \| \| **0** \| **1** \| **2** \| **3** \| **4** \| **5** \| \| **0** \| **1** \| **2** \| **3** \| **4** \| **5** \| \| \| \| --- \| --- \| --- \| --- \| --- \| --- \| --- \| --- \| --- \| --- \| --- \| --- \| --- \| --- \| --- \| --- \| --- \| --- \| --- \| --- \| --- \| --- \| --- \| --- \| --- \| --- \| --- \| --- \| --- \| --- \| --- \| --- \| --- \| --- \| \|  \| \|  \| |
| 16 | Trust | \| 16.1. Challenged  16.2. Suspicious  16.3. Cautious  16.4. Vigilant  16.5. Confident \| \| **0** \| **1** \| **2** \| **3** \| **4** \| **5** \| **______** \| \| --- \| --- \| --- \| --- \| --- \| --- \| --- \| \| **0** \| **1** \| **2** \| **3** \| **4** \| **5** \| \| **0** \| **1** \| **2** \| **3** \| **4** \| **5** \| \| **0** \| **1** \| **2** \| **3** \| **4** \| **5** \| \| **0** \| **1** \| **2** \| **3** \| **4** \| **5** \| \| \| \| --- \| --- \| --- \| --- \| --- \| --- \| --- \| --- \| --- \| --- \| --- \| --- \| --- \| --- \| --- \| --- \| --- \| --- \| --- \| --- \| --- \| --- \| --- \| --- \| --- \| --- \| --- \| --- \| --- \| --- \| --- \| --- \| --- \| --- \| \|  \| \|  \| |
| 17 | Temperament | \| 17.1. Nervous  17.2. agitated  17.3. centered  17.4. Serene  17.5. Pacific \| \| **0** \| **1** \| **2** \| **3** \| **4** \| **5** \| **______** \| \| --- \| --- \| --- \| --- \| --- \| --- \| --- \| \| **0** \| **1** \| **2** \| **3** \| **4** \| **5** \| \| **0** \| **1** \| **2** \| **3** \| **4** \| **5** \| \| **0** \| **1** \| **2** \| **3** \| **4** \| **5** \| \| **0** \| **1** \| **2** \| **3** \| **4** \| **5** \| \| \| \| --- \| --- \| --- \| --- \| --- \| --- \| --- \| --- \| --- \| --- \| --- \| --- \| --- \| --- \| --- \| --- \| --- \| --- \| --- \| --- \| --- \| --- \| --- \| --- \| --- \| --- \| --- \| --- \| --- \| --- \| --- \| --- \| --- \| --- \| \|  \| \| |
| 18 | Character | \| 18.1. Introvert  18.2. Shy  18.3. Reserved  18.4. Curious  18.5. Extroverted \| \| **0** \| **1** \| **2** \| **3** \| **4** \| **5** \| **______** \| \| --- \| --- \| --- \| --- \| --- \| --- \| --- \| \| **0** \| **1** \| **2** \| **3** \| **4** \| **5** \| \| **0** \| **1** \| **2** \| **3** \| **4** \| **5** \| \| **0** \| **1** \| **2** \| **3** \| **4** \| **5** \| \| **0** \| **1** \| **2** \| **3** \| **4** \| **5** \| \| \| \| --- \| --- \| --- \| --- \| --- \| --- \| --- \| --- \| --- \| --- \| --- \| --- \| --- \| --- \| --- \| --- \| --- \| --- \| --- \| --- \| --- \| --- \| --- \| --- \| --- \| --- \| --- \| --- \| --- \| --- \| --- \| --- \| --- \| --- \| \|  \| \|  \| |
| 19 | Awareness | \| 19.1. Unconscious  19.2. Uninformed  19.3. Conscious  19.4. Informed  19.5. Aware \| \| **0** \| **1** \| **2** \| **3** \| **4** \| **5** \| **______** \| \| --- \| --- \| --- \| --- \| --- \| --- \| --- \| \| **0** \| **1** \| **2** \| **3** \| **4** \| **5** \| \| **0** \| **1** \| **2** \| **3** \| **4** \| **5** \| \| **0** \| **1** \| **2** \| **3** \| **4** \| **5** \| \| **0** \| **1** \| **2** \| **3** \| **4** \| **5** \| \| \| \| --- \| --- \| --- \| --- \| --- \| --- \| --- \| --- \| --- \| --- \| --- \| --- \| --- \| --- \| --- \| --- \| --- \| --- \| --- \| --- \| --- \| --- \| --- \| --- \| --- \| --- \| --- \| --- \| --- \| --- \| --- \| --- \| --- \| --- \| \|  \| \|  \| |
| 20 | Instinctiveness  (Ego-Ex) | \| 20.1. Visceral  20.2. Emotional  20.3. Measured  20.4. Meditative  20.5. Mental \| \| \| **0** \| **1** \| **2** \| **3** \| **4** \| **5** \| **______** \| \| --- \| --- \| --- \| --- \| --- \| --- \| --- \| \| **0** \| **1** \| **2** \| **3** \| **4** \| **5** \| \| **0** \| **1** \| **2** \| **3** \| **4** \| **5** \| \| **0** \| **1** \| **2** \| **3** \| **4** \| **5** \| \| **0** \| **1** \| **2** \| **3** \| **4** \| **5** \| \| \| --- \| --- \| --- \| --- \| --- \| --- \| --- \| --- \| --- \| --- \| --- \| --- \| --- \| --- \| --- \| --- \| --- \| --- \| --- \| --- \| --- \| --- \| --- \| --- \| --- \| --- \| --- \| --- \| --- \| --- \| --- \| --- \| --- \| --- \| \|  \|  \| \| |
| 21 | Stability (Strength)  of the Ego | \| 21.1. Unstable  21.2. Precarious  21.3. Balanced  21.4. Reasonable  21.5. Stable \| \| **0** \| **1** \| **2** \| **3** \| **4** \| **5** \| **______** \| \| --- \| --- \| --- \| --- \| --- \| --- \| --- \| \| **0** \| **1** \| **2** \| **3** \| **4** \| **5** \| \| **0** \| **1** \| **2** \| **3** \| **4** \| **5** \| \| **0** \| **1** \| **2** \| **3** \| **4** \| **5** \| \| **0** \| **1** \| **2** \| **3** \| **4** \| **5** \| \| \| \| --- \| --- \| --- \| --- \| --- \| --- \| --- \| --- \| --- \| --- \| --- \| --- \| --- \| --- \| --- \| --- \| --- \| --- \| --- \| --- \| --- \| --- \| --- \| --- \| --- \| --- \| --- \| --- \| --- \| --- \| --- \| --- \| --- \| --- \| \|  \| \|  \| |
| 22 | Adaptation | \| 22.1. Conservative  22.2. Pragmatic  22.3. Adequate  22.4. Assertive  22.5. Liberal \| \| **0** \| **1** \| **2** \| **3** \| **4** \| **5** \| **______** \| \| --- \| --- \| --- \| --- \| --- \| --- \| --- \| \| **0** \| **1** \| **2** \| **3** \| **4** \| **5** \| \| **0** \| **1** \| **2** \| **3** \| **4** \| **5** \| \| **0** \| **1** \| **2** \| **3** \| **4** \| **5** \| \| **0** \| **1** \| **2** \| **3** \| **4** \| **5** \| \| \| \| --- \| --- \| --- \| --- \| --- \| --- \| --- \| --- \| --- \| --- \| --- \| --- \| --- \| --- \| --- \| --- \| --- \| --- \| --- \| --- \| --- \| --- \| --- \| --- \| --- \| --- \| --- \| --- \| --- \| --- \| --- \| --- \| --- \| --- \| \|  \| \|  \| |
| 23 | Opening | \| 23.1. Closed  23.2. Committed  23.3. Resilient  23.4. Disengaged  23.5. Open \| \| **0** \| **1** \| **2** \| **3** \| **4** \| **5** \| **______** \| \| --- \| --- \| --- \| --- \| --- \| --- \| --- \| \| **0** \| **1** \| **2** \| **3** \| **4** \| **5** \| \| **0** \| **1** \| **2** \| **3** \| **4** \| **5** \| \| **0** \| **1** \| **2** \| **3** \| **4** \| **5** \| \| **0** \| **1** \| **2** \| **3** \| **4** \| **5** \| \| \| \| --- \| --- \| --- \| --- \| --- \| --- \| --- \| --- \| --- \| --- \| --- \| --- \| --- \| --- \| --- \| --- \| --- \| --- \| --- \| --- \| --- \| --- \| --- \| --- \| --- \| --- \| --- \| --- \| --- \| --- \| --- \| --- \| --- \| --- \| \|  \| \|  \| |
| 24 | Self-efficacy | \| 24.1 Ineffective  24.2. Inefficient  24.3. Orderly  24.4. Efficient  24.5. Effective \| \| **0** \| **1** \| **2** \| **3** \| **4** \| **5** \| **______** \| \| --- \| --- \| --- \| --- \| --- \| --- \| --- \| \| **0** \| **1** \| **2** \| **3** \| **4** \| **5** \| \| **0** \| **1** \| **2** \| **3** \| **4** \| **5** \| \| **0** \| **1** \| **2** \| **3** \| **4** \| **5** \| \| **0** \| **1** \| **2** \| **3** \| **4** \| **5** \| \| \| \| --- \| --- \| --- \| --- \| --- \| --- \| --- \| --- \| --- \| --- \| --- \| --- \| --- \| --- \| --- \| --- \| --- \| --- \| --- \| --- \| --- \| --- \| --- \| --- \| --- \| --- \| --- \| --- \| --- \| --- \| --- \| --- \| --- \| --- \| \|  \| \|  \| |

**Supporting information 9**: Item-dysfunctional assignment codes in Perrotta Integrative Clinical Interviews - PICI-TA-3

| ***N*** | ***Type of dysfunctional structure*** | ***Reference items***   \| *Dysfunctional traits* \| \| \| \| \| \| \| \| \| \| --- \| --- \| --- \| --- \| --- \| --- \| --- \| --- \| --- \| \| **I** \| **II** \| **III** \| **IV** \| **V** \| **VI** \| **VII** \| **VIII** \| **IX** \| |
| --- | --- | --- | --- | --- | --- | --- | --- | --- | --- | --- | --- | --- | --- | --- | --- | --- | --- | --- | --- | --- |
| **1** | ANXIOUS | \| 1 \| 2 \| 3-4 \| 5 \| 6-7 \| 8 \| 9 \| 10-11 \| 12 \| \| --- \| --- \| --- \| --- \| --- \| --- \| --- \| --- \| --- \| |
| **2** | FOBIC | \| 2 \| 9 \| 10-11 \| 12 \| 13 \| 14 \| 15 \| 16 \| 17 \| \| --- \| --- \| --- \| --- \| --- \| --- \| --- \| --- \| --- \| |
| **3** | AVITANT | \| 18 \| 19 \| 20 \| 21 \| 22 \| 23 \| 24-25-26 \| 27 \| 28 \| \| --- \| --- \| --- \| --- \| --- \| --- \| --- \| --- \| --- \| |
| **4** | OBSESSIVE | \| 2 \| 29 \| 30 \| 31 \| 32 \| 33 \| 34 \| 35 \| 36 \| \| --- \| --- \| --- \| --- \| --- \| --- \| --- \| --- \| --- \| |
| **5** | SOMATIC | \| 3-4 \| 8 \| 9 \| 21 \| 29 \| 34 \| 36 \| 37 \| 38 \| \| --- \| --- \| --- \| --- \| --- \| --- \| --- \| --- \| --- \| |
| **6** | MANIACAL | \| 12 \| 36 \| 39 \| 40 \| 41 \| 42 \| 43-44-45 \| 46 \| 47-48 \| \| --- \| --- \| --- \| --- \| --- \| --- \| --- \| --- \| --- \| |
| **7** | BIPOLAR | \| 6-7 \| 8 \| 39 \| 49 \| 50 \| 51 \| 52-53 \| 54 \| 55 \| \| --- \| --- \| --- \| --- \| --- \| --- \| --- \| --- \| --- \| |
| **8** | EMOTIONAL | \| 8 \| 53 \| 56-63 \| 57 \| 58 \| 59 \| 60 \| 61 \| 62 \| \| --- \| --- \| --- \| --- \| --- \| --- \| --- \| --- \| --- \| |
| **9** | DEPENDENT | \| 20 \| 21 \| 24-25-26 \| 64 \| 65 \| 66 \| 67 \| 68 \| 69 \| \| --- \| --- \| --- \| --- \| --- \| --- \| --- \| --- \| --- \| |
| **10** | DEPRESSIVE | \| 7 \| 9 \| 53 \| 70 \| 71-72 \| 73 \| 74 \| 75 \| 76 \| \| --- \| --- \| --- \| --- \| --- \| --- \| --- \| --- \| --- \| |
| **11** | BORDERLINE | \| 39 \| 49 \| 52-53 \| 60 \| 77-78 \| 79 \| 80 \| 81-82 \| 83 \| \| --- \| --- \| --- \| --- \| --- \| --- \| --- \| --- \| --- \| |
| **12** | ISTRIONICS | \| 77-78 \| 85 \| 86 \| 87-88 \| 89 \| 90 \| 91 \| 92-93 \| 94 \| \| --- \| --- \| --- \| --- \| --- \| --- \| --- \| --- \| --- \| |
| **13** | NARCISSIST-OVERT | \| 52 \| 62 \| 95 \| 96-97 \| 98 \| 99 \| 100 \| 101 \| 102 \| \| --- \| --- \| --- \| --- \| --- \| --- \| --- \| --- \| --- \| |
| **14** | NARCISSIST-COVERT | \| 32 \| 37 \| 38 \| 84 \| 103 \| 104 \| 105 \| 106 \| 107 \| \| --- \| --- \| --- \| --- \| --- \| --- \| --- \| --- \| --- \| |
| **15** | ANTISOCIAL | \| 52 \| 56-63 \| 60-61 \| 62 \| 76 \| 81-82 \| 95 \| 108 \| 109 \| \| --- \| --- \| --- \| --- \| --- \| --- \| --- \| --- \| --- \| |
| **16** | SADIC | \| 53 \| 76 \| 108 \| 110 \| 111 \| 112 \| 113 \| 114 \| 115-116 \| \| --- \| --- \| --- \| --- \| --- \| --- \| --- \| --- \| --- \| |
| **17** | MASOCHIST | \| 49 \| 53 \| 117 \| 118 \| 119 \| 120 \| 121 \| 122 \| 123 \| \| --- \| --- \| --- \| --- \| --- \| --- \| --- \| --- \| --- \| |
| **18** | PSYCHOPATHIC | \| 52-53 \| 60-61 \| 95 \| 108 \| 109 \| 124 \| 125-126 \| 127 \| 128 \| \| --- \| --- \| --- \| --- \| --- \| --- \| --- \| --- \| --- \| |
| **19** | SCHIZOFRENIC | \| 128 \| 129 \| 130-131 \| 132 \| 133 \| 134 \| 135 \| 136 \| 137 \| \| --- \| --- \| --- \| --- \| --- \| --- \| --- \| --- \| --- \| |
| **20** | SCHIZOID | \| 128 \| 131 \| 138 \| 139 \| 140-141 \| 142 \| 143 \| 144 \| 145 \| \| --- \| --- \| --- \| --- \| --- \| --- \| --- \| --- \| --- \| |
| **21** | SCHIZOTIPIC | \| 128 \| 137 \| 146 \| 147 \| 148 \| 149 \| 150 \| 151 \| 152 \| \| --- \| --- \| --- \| --- \| --- \| --- \| --- \| --- \| --- \| |
| **22** | SCHIZOAFFECTIVE | \| 8 \| 39 \| 128 \| 136 \| 137 \| 153 \| 154 \| 155 \| 156 \| \| --- \| --- \| --- \| --- \| --- \| --- \| --- \| --- \| --- \| |
| **23** | DELIRANT | \| 37 \| 63 \| 128 \| 146 \| 153 \| 154 \| 157 \| 158 \| 159 \| \| --- \| --- \| --- \| --- \| --- \| --- \| --- \| --- \| --- \| |
| **24** | PARANOIC | \| 76 \| 157 \| 160 \| 161 \| 162 \| 163 \| 164 \| 165 \| 166 \| \| --- \| --- \| --- \| --- \| --- \| --- \| --- \| --- \| --- \| |
| **25** | DISSOCIATIVE | \| 8 \| 37 \| 167 \| 168 \| 169 \| 170 \| 171 \| 172 \| 173 \| \| --- \| --- \| --- \| --- \| --- \| --- \| --- \| --- \| --- \| |

**Supporting information 10**: Item-dysfunctional assignment codes in Perrotta Integrative Clinical Interviews - PICI-C-3

| ***N*** | ***Type of dysfunctional structure*** | ***Reference items***   \| *Dysfunctional traits* \| \| \| \| \| \| \| \| \| \| --- \| --- \| --- \| --- \| --- \| --- \| --- \| --- \| --- \| \| **I** \| **II** \| **III** \| **IV** \| **V** \| **VI** \| **VII** \| **VIII** \| **IX** \| |
| --- | --- | --- | --- | --- | --- | --- | --- | --- | --- | --- | --- | --- | --- | --- | --- | --- | --- | --- | --- | --- |
| **1** | ANXIOUS | \| 1 \| 2 \| 3-4 \| 5 \| 6-7 \| 8 \| 9 \| 10-11 \| 12 \| \| --- \| --- \| --- \| --- \| --- \| --- \| --- \| --- \| --- \| |
| **2** | FOBIC | \| 2 \| 9 \| 10-11 \| 12 \| 13 \| 14 \| 15 \| 16 \| 17 \| \| --- \| --- \| --- \| --- \| --- \| --- \| --- \| --- \| --- \| |
| **3** | AVITANT | \| 18 \| 19 \| 20 \| 21 \| 22 \| 23 \| 24-25-26 \| 27 \| 28 \| \| --- \| --- \| --- \| --- \| --- \| --- \| --- \| --- \| --- \| |
| **4** | OBSESSIVE | \| 2 \| 29 \| 30 \| 31 \| 32 \| 33 \| 34 \| 35 \| 36 \| \| --- \| --- \| --- \| --- \| --- \| --- \| --- \| --- \| --- \| |
| **5** | SOMATIC | \| 3-4 \| 8 \| 9 \| 21 \| 29 \| 34 \| 36 \| 37 \| 38 \| \| --- \| --- \| --- \| --- \| --- \| --- \| --- \| --- \| --- \| |
| **6** | MANIACAL | \| 12 \| 36 \| 39 \| 40 \| 41 \| 42 \| 43-44-45 \| 46 \| 47-48 \| \| --- \| --- \| --- \| --- \| --- \| --- \| --- \| --- \| --- \| |
| **7** | BIPOLAR | \| 6-7 \| 8 \| 39 \| 49 \| 50 \| 51 \| 52-53 \| 54 \| 55 \| \| --- \| --- \| --- \| --- \| --- \| --- \| --- \| --- \| --- \| |
| **8** | DISREGULANT | \| 6 \| 8 \| 56 \| 57 \| 58 \| 59 \| 60 \| 61 \| 62 \| \| --- \| --- \| --- \| --- \| --- \| --- \| --- \| --- \| --- \| |
| **9** | INSECURE | \| 8 \| 56 \| 58 \| 59 \| 63 \| 64 \| 65 \| 66 \| 67-68 \| \| --- \| --- \| --- \| --- \| --- \| --- \| --- \| --- \| --- \| |
| **10** | OPPOSITIVE | \| 6 \| 8 \| 47 \| 57 \| 58 \| 59 \| 69 \| 70 \| 71 \| \| --- \| --- \| --- \| --- \| --- \| --- \| --- \| --- \| --- \| |
| **11** | AGGRESSIVE | \| 6 \| 8 \| 57 \| 59 \| 61 \| 71 \| 72 \| 73 \| 74 \| \| --- \| --- \| --- \| --- \| --- \| --- \| --- \| --- \| --- \| |
| **12** | THEATRICAL | \| 39 \| 75 \| 76 \| 77 \| 78 \| 79 \| 80 \| 81 \| 82 \| \| --- \| --- \| --- \| --- \| --- \| --- \| --- \| --- \| --- \| |
| **13** | INHIBIT | \| 33 \| 83 \| 84 \| 85 \| 86 \| 87 \| 88 \| 89 \| 90 \| \| --- \| --- \| --- \| --- \| --- \| --- \| --- \| --- \| --- \| |
| **14** | DISINIBITED | \| 91 \| 92 \| 93 \| 94 \| 95 \| 96 \| 97 \| 98 \| 99 \| \| --- \| --- \| --- \| --- \| --- \| --- \| --- \| --- \| --- \| |
| **15** | DEPENDENT | \| 8 \| 20 \| 21 \| 100 \| 101 \| 102 \| 103 \| 104 \| 105 \| \| --- \| --- \| --- \| --- \| --- \| --- \| --- \| --- \| --- \| |
| **16** | DEPRESSIVE | \| 7 \| 8-53 \| 9 \| 106 \| 107-108 \| 109 \| 110 \| 111 \| 112 \| \| --- \| --- \| --- \| --- \| --- \| --- \| --- \| --- \| --- \| |
| **17** | EGOISTIC | \| 1 \| 49 \| 52 \| 113 \| 114 \| 115 \| 116 \| 117 \| 118 \| \| --- \| --- \| --- \| --- \| --- \| --- \| --- \| --- \| --- \| |
| **18** | LIBIDIC | \| 8 \| 52-53 \| 89 \| 113 \| 119 \| 120 \| 121 \| 122 \| 123 \| \| --- \| --- \| --- \| --- \| --- \| --- \| --- \| --- \| --- \| |
| **19** | PSYCHOTIC | \| 89 \| 113 \| 115 \| 122 \| 124 \| 125 \| 126 \| 127 \| 128 \| \| --- \| --- \| --- \| --- \| --- \| --- \| --- \| --- \| --- \| |

**Supporting information 11**: Scoring sheet for the Perrotta Integrative Clinical Interviews - PICI-FT-3

| **PERSONALITY TYPE** | **STYLES/TRAITS OF BEHAVIOR** | **SUBSCORE** | **FINAL SCORE** |
| --- | --- | --- | --- |
| EMOTIONAL | 1.1 Courage (Timorous)  1.2. Courage (Doubting)  2.4. Intrepid (Sincere)  2.5. Intrapreneurship (Direct)  5.1. Security (Insecure)  5.2. Confidence (Uncertain)  9.3. Sensitivity (Sensitive)  9.4. Sensitivity (Concerned)  9.5. Sensitivity (Hypersensitive)  11.1. Relatedness (Superficial)  11.2. Relationality (Volatile)  12.1. Affectivity (Dependent)  12.2. Affectivity (Subordinate)  13.1 Altruism (Selfish)  13.2. Altruism (Defensive)  16.1. Trust (Challenged)  16.2. Trust (Suspicious)  17.1. Temperament (Nervous)  17.2. Temperament (Agitated)  18.1. Character (Introverted)  18.2. Character (Shy)  19.1. Awareness (Unconscious)  19.2. Awareness (Uninformed)  20.2. Instinct (Emotional)  21.1. Ego stability (Unstable)  21.2. Ego Stability (Precarious) | _____ / 5  _____ / 5  _____ / 5  _____ / 5  _____ / 5  _____ / 5  _____ / 5  _____ / 5  _____ / 5  _____ / 5  _____ / 5  _____ / 5  _____ / 5  _____ / 5  _____ / 5  _____ / 5  _____ / 5  _____ / 5  _____ / 5  _____ / 5  _____ / 5  _____ / 5  _____ / 5  _____ / 5  _____ / 5  _____ / 5 | ________ / **130**  0-40 : Weakly emotional  41-80: Averagely emotional  81-130: Markedly emotional |
| RATIONAL | 1.1. Courage (Safe)  2.3. Intrapreneurship (Clear)  3.3. Self-control (Diligent)  4.3. Energy (Harmonious)  5.3. Safety (Responsible)  6.3. Dexterity (Careful)  10.1. Emotionality (Transparent)  10.2. Emotionality (Limpid)  10.3. Emotionality (Adapted)  11.3. Relatability (Reliable)  12.3. Affectivity (Autonomous)  13.3. Altruism (Available)  14.3. Imagination (Symmetrical)  15.3. Morality (Cooperative)  16.3. Confidence (Cautious)  17.3. Temperament (Centered)  19.3. Awareness (Conscious)  20.5. Instinct (Mental)  21.3. Ego stability (Balanced)  22.3. Adaptation (Adequate)  23.3. Openness (Resilient)  24.3. Self-efficacy (Orderly) | _____ / 5  _____ / 5  _____ / 5  _____ / 5  _____ / 5  _____ / 5  _____ / 5  _____ / 5  _____ / 5  _____ / 5  _____ / 5  _____ / 5  _____ / 5  _____ / 5  _____ / 5  _____ / 5  _____ / 5  _____ / 5  _____ / 5  _____ / 5  _____ / 5  _____ / 5 | ________ / **110**  0-35 : Weakly rational  36-70: Averagely rational  71-110: Markedly rational |
| INSTINCTIVE | 1.1 Courage (Adventurous)  1.2. Courage (Fearless)  3.4. Self-control (Free)  3.5. Self-control (Hypocontrolling)  14.1 Imagination (Abstract)  14.2. Fantasy (Artistic)  15.1. Morality (Transgressive)  15.2. Morality (Alternative)  20.1. Instinct (Visceral) | _____ / 5  _____ / 5  _____ / 5  _____ / 5  _____ / 5  _____ / 5  _____ / 5  _____ / 5  _____ / 5 | ________ / **45**  0-15 : Weakly instinctive  16-30: Averagely instinctive  31-45: Markedly instinctive |
| DYNAMIC | 2.1. Intrapreneurship (Cheeky)  2.2. Intrapreneurship (Spontaneous)  4.4. Energy (Energetic)  4.5. Energy (Active)  6.4. Dexterity (Cunning)  6.5. Dexterity (Shrewd)  7.3. Sociability (Sociable)  7.4. Sociability (Friendly)  7.5. Sociability (Cozy)  8.3. Passionality (Engaging)  8.4. Passionality (Passionate)  8.5. Passionality (Fiery)  12.4. Affectivity (Independent)  12.5. Affectivity (Individualist)  13.4. Altruism (Good)  13.5. Altruism (Generous)  15.4. Morality (Collaborative)  15.5. Morality (Compliant)  16.4. Trust (Vigilant)  16.5. Confidence (Confident)  18.4. Character (Curious)  18.5. Character (Extroverted)  24.4. Self-efficacy (Efficient)  24.5. Self-efficacy (Effective) | _____ / 5  _____ / 5  _____ / 5  _____ / 5  _____ / 5  _____ / 5  _____ / 5  _____ / 5  _____ / 5  _____ / 5  _____ / 5  _____ / 5  _____ / 5  _____ / 5  _____ / 5  _____ / 5  _____ / 5  _____ / 5  _____ / 5  _____ / 5  _____ / 5  _____ / 5  _____ / 5  _____ / 5 | ________ / **120**  0-40 : Weakly dynamic  41-60: Averagely dynamic  61-120: Markedly dynamic |
| FLEMMATIC | 3.1. Self-control (Hypercontrol)  3.2. Self-control (Moderate)  4.1. Energy (Passive)  4.2. Energy (Tiepid)  6.1. Dexterity (Unprovided)  6.2. Dexterity (Naive)  7.1. Sociability (Stiff)  7.2. Sociability (Accommodating)  8.1. Passionateness (Indifferent)  8.2. Passionality (Detached)  9.1. Sensitivity (Hyposensitive)  9.2. Sensitivity (Disinterested)  10.4. Emotionality (Protected)  10.5. Emotionality (Opaque)  17.4. Temperament (Serene)  17.5. Temperament (Peaceful)  18.3. Character (Reserved)  20.3. Instinct (Measured)  23.1. Openness (Closed)  23.2. Openness (Committed)  24.1. Self-efficacy (Ineffective)  24.2. Self-efficacy (Ineffective) | _____ / 5  _____ / 5  _____ / 5  _____ / 5  _____ / 5  _____ / 5  _____ / 5  _____ / 5  _____ / 5  _____ / 5  _____ / 5  _____ / 5  _____ / 5  _____ / 5  _____ / 5  _____ / 5  _____ / 5  _____ / 5  _____ / 5  _____ / 5  _____ / 5  _____ / 5 | ________ / **110**  0-35 : Weakly flemmatic  36-70: Averagely flemmatic  71-110: Markedly flemmatic |
| REFLECTIVE | 5.4. Security (Reliable)  5.5. Security (Safe)  11.4. Reliability (Methodical)  11.5. Relatability (Rigid)  14.4. Fantasy (Methodical)  14.5. Fantasy (Concrete)  19.4. Awareness (Informed)  19.5. Awareness (Aware)  20.4. Instinct (Meditative)  21.4. Ego Stability (Reasonable)  21.5. Stability of the Ego (Stable)  22.1. Adaptation (Conservative)  22.2. Adaptation (Pragmatic)  22.4. Adaptation (Assertive)  22.5. Adaptation (Liberal)  23.4. Openness (Disengaged)  23.5. Openness (Open) | _____ / 5  _____ / 5  _____ / 5  _____ / 5  _____ / 5  _____ / 5  _____ / 5  _____ / 5  _____ / 5  _____ / 5  _____ / 5  _____ / 5  _____ / 5  _____ / 5  _____ / 5  _____ / 5  _____ / 5 | ________ / **85**  0-29 : Weakly reflective  30-59: Averagely reflective  60-85: Markedly reflective |

**Supporting information 12**: Reference values for scoring personality types in the Perrotta Integrative Clinical Interviews - PICI-FT-3. Red zone: poor utilization of the specific functional personality type. Orange zone: uncommon utilization of the specific functional personality type. Yellow zone: well-balanced utilization of the specific functional personality type. Green zone: marked utilization of the specific functional personality typology. Purple zone: prevalent utilization of the specific functional personality typology.

| PERSONALITY TYPE: **EMOTIONAL** | | | | | | | | | | | | |
| --- | --- | --- | --- | --- | --- | --- | --- | --- | --- | --- | --- | --- |
|  |  |  |  |  |  |  |  |  |  |  |  |  |
| **0-10** | **11-20** | **21-30** | **31-40** | **41-50** | **51-60** | **61-70** | **71-80** | **81-90** | **91-100** | **101-110** | **111-120** | **121-130** |
| PERSONALITY TYPE: **RATIONAL** | | | | | | | | | | | | |
|  |  |  |  |  |  |  |  |  |  |  |  |  |
| **0-8** | **9-16** | **17-24** | **25-32** | **33-40** | **41-48** | **49-62** | **63-70** | **71-78** | **79-86** | **87-94** | **95-102** | **103-110** |
| PERSONALITY TYPE: **INSTINCTIVE** | | | | | | | | | | | | |
|  |  |  |  |  |  |  |  |  |  |  |  |  |
| **0-3** | **4-7** | **8-10** | **11-13** | **14-16** | **17-19** | **20-25** | **26-28** | **29-31** | **32-34** | **35-37** | **38-41** | **42-45** |
| PERSONALITY TYPE: **DYNAMIC** | | | | | | | | | | | | |
|  |  |  |  |  |  |  |  |  |  |  |  |  |
| **0-9** | **9-18** | **19-27** | **28-36** | **37-45** | **46-54** | **55-66** | **67-75** | **76-84** | **85-93** | **94-102** | **103-111** | **112-120** |
| PERSONALITY TYPE: **FLEMMATIC** | | | | | | | | | | | | |
|  |  |  |  |  |  |  |  |  |  |  |  |  |
| **0-8** | **9-16** | **17-24** | **25-32** | **33-40** | **41-48** | **49-62** | **63-70** | **71-78** | **79-86** | **87-94** | **95-102** | **103-110** |
| PERSONALITY TYPE: **REFLECTIVE** | | | | | | | | | | | | |
|  |  |  |  |  |  |  |  |  |  |  |  |  |
| **0-6** | **7-12** | **13-19** | **20-26** | **27-33** | **34-40** | **41-48** | **49-54** | **55-60** | **61-67** | **68-73** | **74-79** | **80-85** |

**Supporting information 13**: Reference values for scoring personality types in the Perrotta Integrative Clinical Interviews - PICI-FT-3. Red zone: poor utilization of the specific area. Orange zone: uncommon utilization of the specific area. Yellow zone: well-balanced utilization of the specific area. Green zone: marked utilization of the specific area. Purple zone: prevalent utilization of the specific area.

| **N** | **AREA OF OPERATION** | **Point (p_)** |
| --- | --- | --- |
| 1* | Temperament | / 25 |
| 2* | Courage | / 25 |
| 3* | Resourcefulness | / 25 |
| 4* | Self-control | / 25 |
| 5* | Sensitivity | / 25 |
| 6* | Expression in action | / 25 |
| 7* | Awareness | / 25 |
| 8* | Instinctiveness (Ego-Ex) | / 25 |
| 9* | Affectivity | / 25 |
| 10* | Energy | / 25 |
| 11* | Stability (Strength) of the Ego | / 25 |
| 12* | Security | / 25 |
| 13* | Relationality (relationships with others) | / 25 |
| 14* | Emotionality | / 25 |
| 15* | Dexterity | / 25 |
| 16* | Adaptation | / 25 |
| 17* | Sociability | / 25 |
| 18* | Altruism | / 25 |
| 19* | Openness | / 25 |
| 20* | Passionately | / 25 |
| 21* | Imagination | / 25 |
| 22* | Morality | / 25 |
| 23* | Confidence | / 25 |
| 24* | Self-efficacy | / 25 |

**Supporting information 14**: Grid associated with the reference values for scoring personality types in the Perrotta Integrative Clinical Interviews - PICI-FT-3. Red zone: weak functioning of the specific area. Orange zone: mediocre functioning of the specific area. Yellow zone: balanced functioning of the specific area. Green zone: consistent functioning of the specific area. Purple zone: robust functioning of the specific area.

| **24*** |  |  |  |  |  |  |  |  |  |  |  |  |  |  |  |  |  |  |  |  |  |  |  |  |  |  |
| --- | --- | --- | --- | --- | --- | --- | --- | --- | --- | --- | --- | --- | --- | --- | --- | --- | --- | --- | --- | --- | --- | --- | --- | --- | --- | --- |
| **23*** |  |  |  |  |  |  |  |  |  |  |  |  |  |  |  |  |  |  |  |  |  |  |  |  |  |  |
| **22*** |  |  |  |  |  |  |  |  |  |  |  |  |  |  |  |  |  |  |  |  |  |  |  |  |  |  |
| **21*** |  |  |  |  |  |  |  |  |  |  |  |  |  |  |  |  |  |  |  |  |  |  |  |  |  |  |
| **20*** |  |  |  |  |  |  |  |  |  |  |  |  |  |  |  |  |  |  |  |  |  |  |  |  |  |  |
| **19*** |  |  |  |  |  |  |  |  |  |  |  |  |  |  |  |  |  |  |  |  |  |  |  |  |  |  |
| **18*** |  |  |  |  |  |  |  |  |  |  |  |  |  |  |  |  |  |  |  |  |  |  |  |  |  |  |
| **17*** |  |  |  |  |  |  |  |  |  |  |  |  |  |  |  |  |  |  |  |  |  |  |  |  |  |  |
| **16*** |  |  |  |  |  |  |  |  |  |  |  |  |  |  |  |  |  |  |  |  |  |  |  |  |  |  |
| **15*** |  |  |  |  |  |  |  |  |  |  |  |  |  |  |  |  |  |  |  |  |  |  |  |  |  |  |
| **14*** |  |  |  |  |  |  |  |  |  |  |  |  |  |  |  |  |  |  |  |  |  |  |  |  |  |  |
| **13*** |  |  |  |  |  |  |  |  |  |  |  |  |  |  |  |  |  |  |  |  |  |  |  |  |  |  |
| **12*** |  |  |  |  |  |  |  |  |  |  |  |  |  |  |  |  |  |  |  |  |  |  |  |  |  |  |
| **11*** |  |  |  |  |  |  |  |  |  |  |  |  |  |  |  |  |  |  |  |  |  |  |  |  |  |  |
| **10*** |  |  |  |  |  |  |  |  |  |  |  |  |  |  |  |  |  |  |  |  |  |  |  |  |  |  |
| **9*** |  |  |  |  |  |  |  |  |  |  |  |  |  |  |  |  |  |  |  |  |  |  |  |  |  |  |
| **8*** |  |  |  |  |  |  |  |  |  |  |  |  |  |  |  |  |  |  |  |  |  |  |  |  |  |  |
| **7*** |  |  |  |  |  |  |  |  |  |  |  |  |  |  |  |  |  |  |  |  |  |  |  |  |  |  |
| **6*** |  |  |  |  |  |  |  |  |  |  |  |  |  |  |  |  |  |  |  |  |  |  |  |  |  |  |
| **5*** |  |  |  |  |  |  |  |  |  |  |  |  |  |  |  |  |  |  |  |  |  |  |  |  |  |  |
| **4*** |  |  |  |  |  |  |  |  |  |  |  |  |  |  |  |  |  |  |  |  |  |  |  |  |  |  |
| **3*** |  |  |  |  |  |  |  |  |  |  |  |  |  |  |  |  |  |  |  |  |  |  |  |  |  |  |
| **2*** |  |  |  |  |  |  |  |  |  |  |  |  |  |  |  |  |  |  |  |  |  |  |  |  |  |  |
| **1*** |  |  |  |  |  |  |  |  |  |  |  |  |  |  |  |  |  |  |  |  |  |  |  |  |  |  |
|  | **0** | **1** | **2** | **3** | **4** | **5** | **6** | **7** | **8** | **9** | **10** | **11** | **12** | **13** | **14** | **15** | **16** | **17** | **18** | **19** | **20** | **21** | **22** | **23** | **24** | **25** |
